# Baseline normalization choices inflate classification performance in wearable health monitoring: quantification and mitigation strategies

**DOI:** 10.3389/fdgth.2026.1827279

**Published:** 2026-08-18

**Authors:** Alessandro Tognotti, Corin F. Otesteanu, Luca Anceschi, Carlo Menon

**Affiliations:** 1 Department of Electronics, Information and Bioengineering, Politecnico di Milano, Milan, Italy; 2 Department of Health Sciences and Technology, ETH Zurich, Zurich, Switzerland

**Keywords:** anxiety detection, digital health, machine learning, physiological signals, signal processing, wearable devices

## Abstract

Wearable health monitoring systems provide continuous physiological measurements with growing use in clinical and population health applications. Baseline normalization enables individual-specific calibration of physiological signals to account for inter-subject variability; however, systematic evaluation of how the temporal relationship between normalization windows and evaluation data influences reported classification performance remains limited. Using physiological anxiety detection as a case study, we investigate this methodological challenge with the PhysioNet Spider Fear dataset (52 participants, including measurements of heart activity, skin conductance, and respiration), comparing normalization approaches and three baseline selection strategies. While maintaining strict subject-independent partitions, we show that including the normalized baseline in the evaluation window (test-inclusive baseline normalization) produced accuracies of 89%–93%, compared with when the baseline window is excluded (test-exclusive baseline normalization), which yielded 77%–85%, a 3–13 percentage-point difference consistent across classifiers, window sizes, and class-balance conditions. Two test-exclusive strategies using physiologically distinct baseline states (rest vs. anxiety) performed equivalently, indicating that performance differences were driven by temporal overlap rather than baseline selection. Full-feature normalization improved balanced accuracy by 11%–18% relative to unnormalized features; however, after removing temporal overlap, performance did not differ between normalization approaches. Prolonged stimulus exposure was associated with a 4–7 percentage-point reduction in balanced accuracy, consistent with habituation effects. Increasing window duration from 10 s to 60 s was associated with higher balanced accuracy (72%–74% vs. 78%–79%), whereas data augmentation did not improve performance under moderate class imbalance. We recommend explicit reporting of baseline temporal boundaries to enable accurate performance comparisons across studies and reliable assessment of clinical applicability in wearable health monitoring.

## 1 Introduction

Anxiety disorders affect approximately 4% of the global population, translating to over 300 million individuals worldwide (1, 2). These conditions impose substantial burdens on healthcare systems and individual quality of life while remaining challenging to diagnose through traditional clinical assessment methods that rely heavily on subjective self-report questionnaires such as the State-Trait Anxiety Inventory (STAI) and periodic clinician observations (3). The development of objective, continuous anxiety monitoring systems through physiological signal analysis holds significant promise for revolutionizing clinical practice by enabling real-time assessment during therapeutic interventions and supporting personalized treatment adaptation.

Recent advances in wearable sensor technology and machine learning have enabled automated anxiety detection from physiological signals. Electrocardiogram (ECG) captures heart rate variability reflecting autonomic nervous system modulation, electrodermal activity (EDA) measures skin conductance changes from sympathetic activation, and respiration (RSP) provides breathing pattern information related to stress responses (4). The field has witnessed remarkable classification performance across diverse experimental paradigms. Ihmig et al. (5) demonstrated the feasibility of anxiety detection using the PhysioNet spider fear dataset, reporting 91% accuracy for binary classification with 60-s windows and 89% with 10-s windows using minimal feature sets extracted from ECG, EDA, and RSP signals. The integration of multiple physiological modalities has demonstrated superior discriminative capability compared to single-signal approaches. Gazi et al. (6) reported substantial performance improvements when combining ECG, EDA, and RSP signals, achieving 85% accuracy in detecting the anxiety state compared to 78% when using only ECG and EDA.

Additional anxiety detection studies have explored various machine learning algorithms, with Support Vector Machines (7), Random Forests (6, 8), and Neural Networks (9, 10) each demonstrating competitive performance. Classification accuracies approaching 98% have been reported, depending on the specific combination of physiological modalities, feature sets, and algorithmic approaches employed (11).

Despite these promising results, fundamental methodological challenges limit the reliability and reproducibility of reported performance. First, *baseline normalization* is widely employed in anxiety detection research to account for inter-individual physiological variability (4, 12), yet most fail to document whether baseline computation periods overlap with evaluation data (13, 14). When normalization statistics are computed using data that subsequently appears in test sets (13), performance estimates can be artificially inflated. Herein, we refer to this as *test-inclusive baseline normalization*, and to its counterpart, where baseline normalization data is not included in the test set, as *test-exclusive baseline normalization*. This form of performance inflation is related to what has been termed normalization leakage in the machine learning literature (15, 16), a type of preprocessing leakage in which normalization parameters are derived from data that subsequently appears in the evaluation set. Unlike conventional train-test leakage, this bias can arise even when subject-independent partitions are strictly maintained and model training remains entirely uncontaminated, as long as normalization and evaluation windows overlap temporally. The magnitude of this bias in wearable health monitoring, and more specifically, physiological anxiety detection, remains unquantified, and the comparative impact of different normalization methods under rigorously controlled, evaluation-independent conditions has not been systematically evaluated. Moreover, *repeated stimulus exposure* during extended exposure protocols may induce habituation, progressively attenuating physiological responses to anxiety-inducing stimuli (17, 18). While exposure therapy literature documents systematic reductions in autonomic reactivity following repeated fear stimulus presentation, the magnitude of this effect on classification performance in physiological anxiety detection remains unquantified. Additionally, *temporal window length* for feature extraction varies widely across studies, ranging from 10 s to 5 min (5). This variation reflects a fundamental trade-off: shorter windows enable near-instantaneous anxiety detection suitable for real-time applications but provide limited samples for computing reliable variability-based statistics (e.g., heart rate variability, respiratory variability), while longer windows improve feature quality but sacrifice near real-time anxiety evaluation. Lastly, *class imbalance* between anxiety and rest samples poses practical challenges for model training. In most anxiety evaluation settings, resting periods are often substantially shorter than anxiety-inducing contexts, creating imbalanced datasets where anxiety samples outnumber rest samples. While data augmentation techniques are commonly employed to address class imbalance (19), their effectiveness for physiological anxiety detection has not been systematically evaluated.

Our study addresses these gaps through a systematic investigation under rigorously controlled experimental conditions. Using the publicly available PhysioNet spider fear dataset (17) to detect anxiety episodes, we pursue four objectives. First, to the best of our knowledge, this is the first study to quantify the extent of performance inflation that arises when baseline normalization periods overlap with test data by comparing three baseline selection strategies: two maintaining strict temporal separation and one intentionally introducing overlap. We evaluate eight normalization methods to determine which transformations are more robust to classification. Second, we assess whether repeated stimulus exposure produces habituation effects that degrade classification performance, comparing early-exposure tasks against extended-exposure tasks to characterize how cumulative stimulus presentation affects physiological discriminability. Third, we characterize how classification accuracy varies with temporal window duration across 10 to 60 s, identifying the window length that optimally balances real-time capability with classification performance. Lastly, we compare three data augmentation strategies to determine whether more complex synthetic sample generation provides measurable benefits over simpler approaches when addressing class imbalance in physiological data.

## 2 Methods

### 2.1 Dataset

This study utilized the spider fear anxiety detection dataset collected by Schäfer et al. (17), and made publicly available through PhysioNet (20). The dataset comprises physiological recordings from 57 spider-fearful individuals aged 18 to 40 years. Participants were recruited and screened for specific phobia diagnostic criteria, requiring a minimum score of 14 on the German Spider Anxiety Screening test and at least 50 points on the Fear of Spiders Questionnaire (5).

The experimental protocol implemented a video-based fear induction paradigm consisting of approximately 35 min of recordings per participant. The session began with a 1-min demonstration clip (clip 0) to familiarize participants with the procedure. Subsequently, participants viewed 16 spider-related video clips (clips 1–16), each lasting 60 s and taken from television documentaries showing detailed shots of spiders. Following the final clip, participants were monitored for five minutes during a resting period to establish individual physiological reference values.

Three physiological modalities were continuously recorded at 100 Hz sampling rate using a BITalino (r)evolution portable biosignal acquisition platform (PLUX—Wireless Biosignals S.A., Lisbon, Portugal) with 10-bit resolution. *Electrocardiogram (ECG)* was captured via three chest electrodes positioned according to standard Lead II configuration for heart rate variability analysis. *Electrodermal activity (EDA)* was measured through two finger electrodes attached to the proximal part of the palm of the participant’s non-dominant hand, reflecting sympathetic nervous system activation. *Respiration (RSP)* was monitored via thoracic belt capturing breathing rate and amplitude variations (5). For each participant, synchronized time-stamped physiological signals were provided along with precise temporal markers indicating the starting and ending timestamps for each video clip and the resting phase.

From the original cohort of 57 participants, data quality was assessed prior to analysis. Quality control procedures included verification of physiological signal integrity, confirmation of trigger information required for precise resting phase identification, and completeness of baseline recordings. Gazi et al. (6) had previously excluded two participants (VP26 and VP70) due to significantly corrupted physiological data. Our assessment revealed additional data integrity issues. Specifically, participant VP41 was excluded due to missing trigger information preventing precise resting phase location, while VP03 and VP08 were excluded due to incomplete baseline recordings (missing most of the resting phase data). Given the dataset’s inherent imbalance toward anxiety-inducing clips (16 min per participant) vs. resting periods (5 min), retaining participants with incomplete resting data would have further compromised minority class representation. After applying these quality criteria, the final analysis dataset comprised 52 participants with complete, high-quality recordings across all three physiological modalities and well-defined temporal segmentation for both anxiety-inducing stimuli and resting baseline periods.

### 2.2 Normalization approaches

Physiological signal normalization relative to individual-specific baselines is essential for robust anxiety classification due to substantial inter-subject variability in autonomic nervous system responses. We evaluated eight normalization methods representing different approaches to handling inter-individual physiological variability. These methods were selected to span the spectrum from simple location shifts to more complex variance-based standardization, following practices established in physiological signal processing literature (5, 12, 21).

*Subtraction* removes the mean baseline value, centering features around zero while preserving scale differences. *Delta* normalization expresses features as ratios relative to baseline, capturing proportional changes. *Relative delta* combines both approaches by computing percentage changes relative to the baseline. *Logarithm* applies logarithmic scaling before the ratio computation, compressing extreme values while preserving sign. *Z-score* standardizes features by removing both location and scale effects through mean subtraction and standard deviation division, assuming Gaussian-distributed features. *Min-max scaling* linearly transforms features to a bounded range based on baseline extrema. *Partial normalization* selectively normalizes only three specific features (ECG MeanNN, RSP MeanNN, EDA Mean) following the approach reported by Ihmig et al. (5), leaving remaining features unnormalized. Finally, *None* provides an unnormalized baseline for comparison. Table 1 summarizes their mathematical formulation, where f represents the feature value, $\mu_{\text{baseline}}$ and $\sigma_{\text{baseline}}$ denote baseline mean and standard deviation, and ϵ is a small constant preventing division by zero.

**Table 1:** Normalization methods evaluated for each baseline strategy.

Method	Formula
Subtraction	$f_{\text{norm}}=f-\mu_{\text{baseline}}$
Delta	$f_{\text{norm}}=\frac{\;f}{\mu_{\text{baseline}}+\epsilon }$
Relative delta	$f_{\text{norm}}=\frac{\;f-\mu_{\text{baseline}}}{\mu_{\text{baseline}}+\epsilon }$
Logarithm	$f_{\text{norm}}=\frac{\text{sgn}(f)\log\;(|f|+\epsilon )}{\text{sgn}(\mu_{\text{baseline}})\log\;(|\mu_{\text{baseline}}|+\epsilon )}$
*Z*-score	$f_{\text{norm}}=\frac{\;f-\mu_{\text{baseline}}}{\sigma_{\text{baseline}}+\epsilon }$
Min-max scaling	$f_{\text{norm}}=\frac{\;f-{\text{min}}_{\text{baseline}}}{{\text{max}}_{\text{baseline}}-{\text{min}}_{\text{baseline}}}$
Partial normalization	Normalizes only ECG MeanNN, RSP MeanNN, EDA Mean (5)
None	No normalization

Normalization statistics were computed independently for each subject by applying the same feature extraction functions described in Section 2.3 to the raw physiological signal of the selected baseline period treated as a single segment. The resulting per-subject statistics were then used to transform feature values extracted from all anxiety and rest windows according to the formulas in Table 1, prior to train/validation/test splitting.

This evaluation framework compares normalization methods across baseline selection strategies to quantify performance differences attributable to normalization choice.

### 2.3 Signal processing and feature extraction

Raw physiological signals underwent standard preprocessing pipelines implemented using the NeuroKit2 library (22) and custom filtering functions. ECG signals were bandpass filtered with cutoff frequencies of 5 and 12 Hz (5, 6) to emphasize QRS complexes, followed by R-peak detection using the Pan-Tompkins algorithm (23) to extract inter-beat intervals. RSP signals were bandpass filtered with cutoff frequencies of 0.1 and 0.4 Hz after edge-padding to isolate the respiratory frequency band, and breath-to-breath intervals were identified via peak detection. EDA signals were low-pass filtered with a 1.5 Hz Butterworth cutoff (5, 6) and decomposed into tonic and phasic components using the NeuroKit2 library, which internally separates the slow-varying tonic level from the fast phasic SCR events, using default parameters. Skin conductance responses were detected automatically through peak detection; from the phasic component, nOR, mmOR, and mdOR were derived, while Mean and SD were computed from the filtered signal to capture tonic activity.

From each preprocessed signal, we extracted a feature set designed to capture statistical, temporal, and nonlinear physiological characteristics. Feature selection was guided by the work of Ihmig et al. (5) combined with established features from Ritsert et al. (24) for ECG and respiration analysis. The complete feature set comprised 27 features distributed across the three modalities as shown in Table 2.

**Table 2:** Extracted physiological features from ECG, RSP, and EDA signals.

ECG (11 features)	RSP (11 features)	EDA (5 features)
Mean	Mean	Mean
SD	SD	SD
NFD	NFD	nOR
NSD	NSD	**mmOR**
MeanNN	MeanNN	**mdOR**
**SDNN**	**SDNN**	
**RMSSD**	**RMSSD**	
**MCVNN**	**MCVNN**	
**SDRMSSD**	**SDRMSSD**	
SD1	SD1	
SD2	SD2	

Features in bold require sufficient inter-beat intervals (SDNN, RMSSD, MCVNN, SDRMSSD) or observed events (mmOR, mdOR) for reliable computation, making them susceptible to missing values in shorter windows.

The extracted features are defined as follows. *Mean* and *SD* represent signal mean and standard deviation. *NFD* and *NSD* denote normalized first and second differences, capturing signal variability. *MeanNN* represents the mean NN interval, where NN denotes normal-to-normal inter-beat intervals. The bolded variability features (*SDNN, RMSSD, MCVNN, SDRMSSD*) represent the standard deviation (SD), root mean square of successive differences, mean coefficient of variation, and SD of RMSSD of NN intervals. They quantify dispersion across successive intervals and require adequate samples for robust estimation; in 10-s windows containing approximately 10–15 cardiac cycles at normal resting heart rate (60–100 bpm) and only 1–4 respiratory cycles at normal breathing rate (6–24 breaths per minute) (5, 6), these features exhibit high uncertainty and are often undefined. *SD1* and *SD2* are Poincaré plot features capturing short-term and long-term variability. For EDA, *nOR* (number of skin conductance responses) counts the number of responses, while *mmOR* (mean magnitude of responses) and *mdOR* (mean duration of responses) represent mean magnitude and mean duration of responses, which are undefined when no complete responses occur within the analysis window. Features requiring a sufficient number of inter-beat intervals, inter-breath intervals, or detected skin conductance responses for reliable estimation (specifically SDNN, RMSSD, MCVNN, SDRMSSD, SD1, and SD2 for both ECG and RSP, and mmOR and mdOR for EDA) are structurally undefined in short analysis windows. Features with more than 30% missing values across samples were removed from the analysis; the remaining features with sporadic missing values were imputed using the class-conditional mean prior to model training.

Prior to model training, we performed feature selection to identify the most discriminative subset of features for anxiety detection using the **minimum Redundancy Maximum Relevance (mRMR)** algorithm (25), which jointly optimizes feature relevance to the prediction target while minimizing inter-feature redundancy. We selected a subset of 15 features and standardized this configuration across all experimental tasks to enable consistent comparisons.

### 2.4 Experimental design

**Figure 1:**
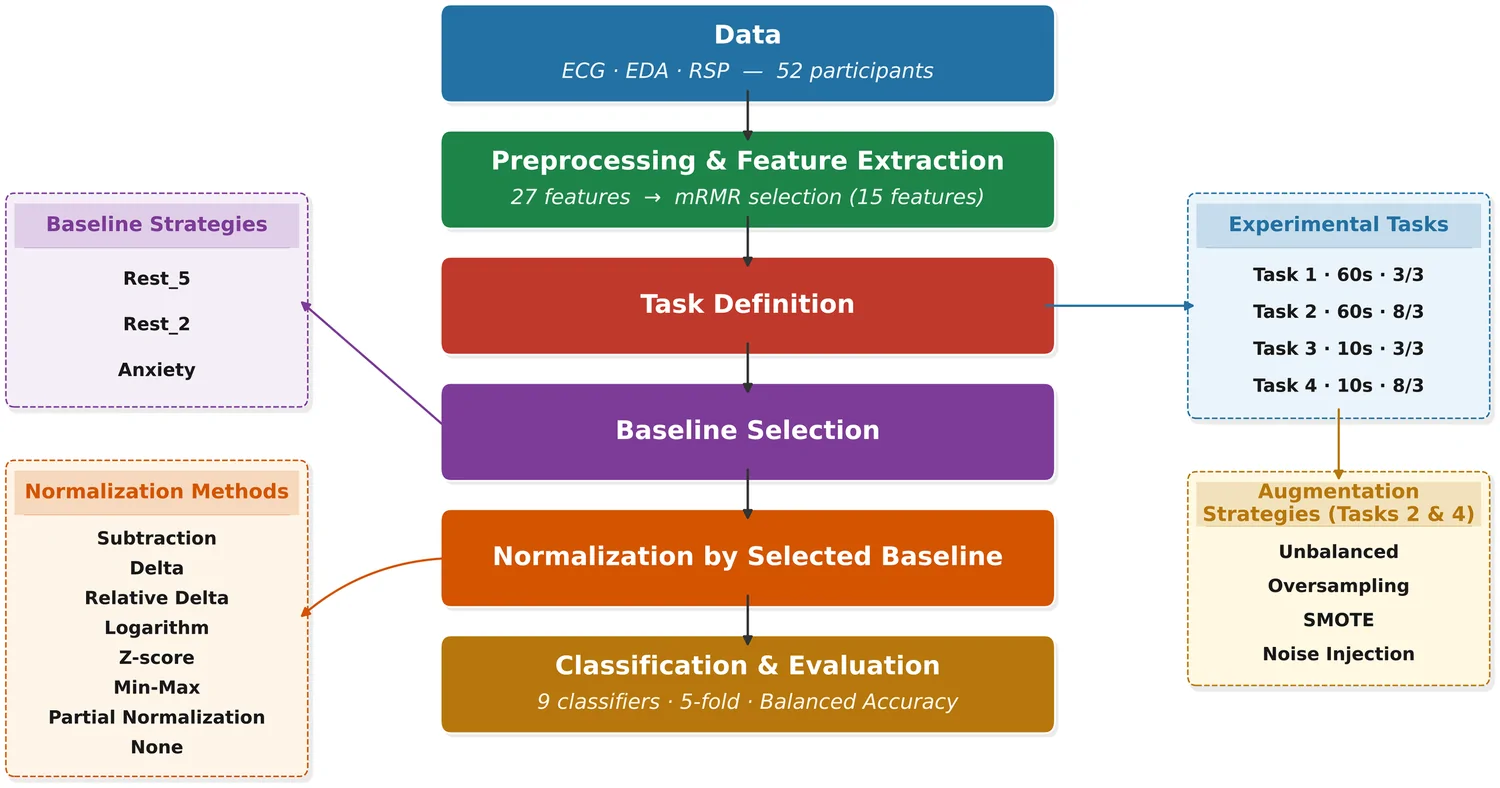
Experimental pipeline. From raw physiological data (ECG, EDA, RSP) to classification, highlighting the four experimental tasks, three baseline selection strategies, eight normalization methods, and augmentation strategies applied in Tasks 2 and 4.

The goal of this study was *binary classification* of anxiety vs. rest states from physiological signals, following the experimental pipeline illustrated in Figure 1.

**Figure 2:**
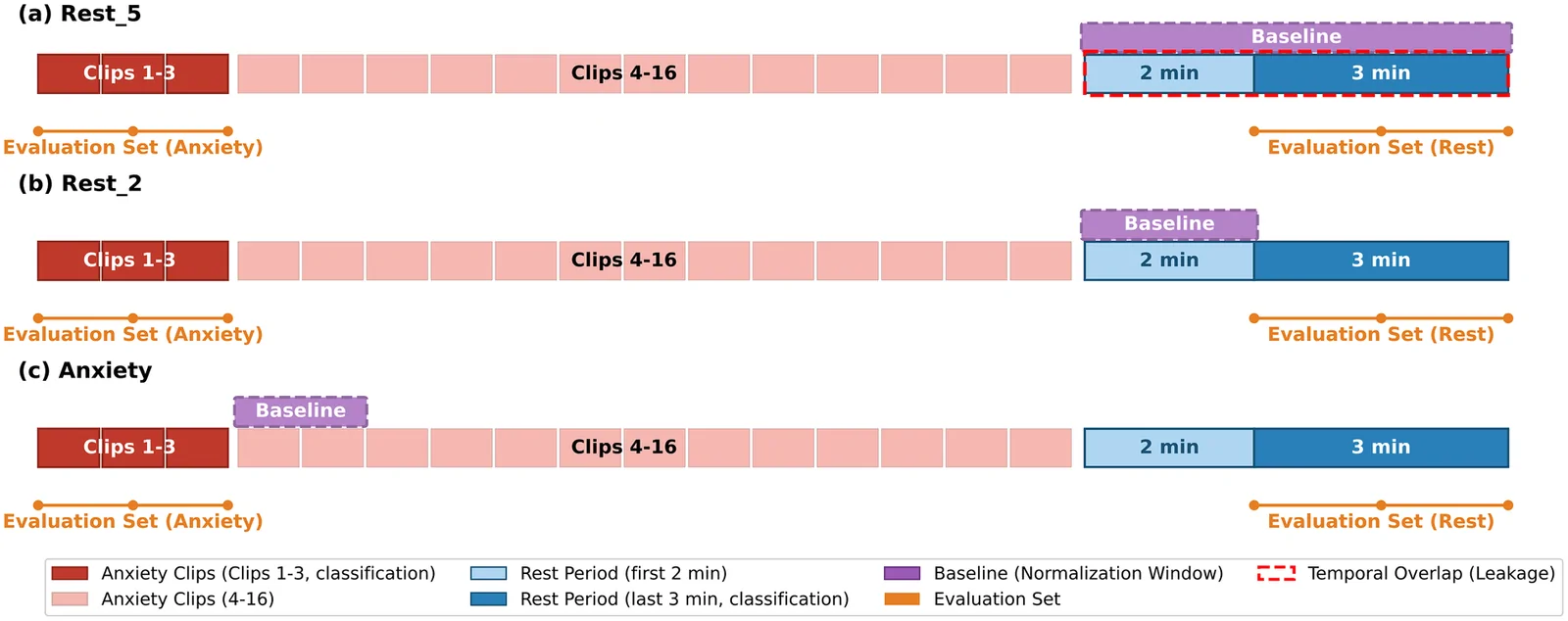
Temporal structure of the three baseline selection strategies in early exposure configuration. **(a)** *Rest_5*: normalization statistics are computed from the entire 5-min resting period (red dashed border), with test-inclusive baseline normalization since the last 3 min of rest overlap with evaluation samples. **(b)** *Rest_2*: only the first 2 min of rest (light blue) are used for normalization, which are entirely excluded from the evaluation set. **(c)** *Anxiety*: normalization is derived from anxiety clips 4–5, temporally disjoint from the classification targets (clips 1–3).

We systematically investigated the impact of baseline normalization and selection strategy on classification performance across combinations of temporal window duration and stimulus-exposure configuration. Three baseline selection strategies were compared, differing in the temporal period used to compute normalization factors. These strategies enabled quantification of performance inflation arising when baseline computation periods overlapped with evaluation samples. Table 3 summarizes their configuration and baseline normalization type. Throughout the Results section, these strategies are referred to as “rest_5”, “rest_2”, and “anxiety”. Figure 2 illustrates the temporal structure of the three baseline selection strategies.

**Table 3:** Summary of baseline selection strategies evaluated across all experimental tasks.

Baseline	Normalization data source	Normalization status
Rest_5	All 5 min of rest	Test-inclusive baseline normalization
Rest_2	First 2 min of rest	Test-exclusive baseline normalization
Anxiety	Anxiety clips following classification targets	Test-exclusive baseline normalization

**Rest_5** computes normalization statistics from the full 5-min rest period, which is included in the evaluation set. Because the baseline window contributes to its own normalization, this self-inclusion can lead to overestimation of model performance.

**Rest_2** represents the most conservative test-exclusive baseline normalization condition, where normalization statistics are computed exclusively from the first two minutes of the resting baseline period. These two minutes are completely excluded from all training and test sets, ensuring zero overlap between normalization data and evaluation samples. This configuration provides an unbiased estimate of performance when baseline statistics must be computed from independent data.

**Anxiety** baseline strategy assesses whether normalizing relative to anxious physiological states provides comparable discrimination to rest-based normalization, while preserving evaluation independence. Rather than relying on a resting period, normalization statistics are derived from anxiety clips that temporally succeed the clips used for classification, thereby ensuring strict separation between normalization and evaluation data. For this reason, anxiety baseline strategy was included as a methodological control condition rather than a clinically deployable approach, as deriving normalization statistics from clips that temporally follow the classification targets precludes prospective deployment.

To investigate habituation effects, we evaluated classification under two exposure subsets. The *early-exposure* subset comprised the first three anxiety clips and the final three minutes of rest, representing conditions where stimuli were relatively novel. The *extended-exposure* subset comprised eight anxiety clips and the same three rest minutes, where repeated stimulus presentation was expected to progressively attenuate physiological responses and reduce class separability. For the anxiety baseline strategy, normalization references were selected from clips temporally succeeding the classification targets to ensure strict evaluation independence: clips 4–5 in early-exposure tasks (Tasks 1 and 3), and clips 9–10 in extended-exposure tasks (Tasks 2 and 4).

For all baseline strategies, normalization statistics were computed independently for each subject using exclusively that subject’s designated baseline window, such that no cross-subject information is involved in the normalization procedure. In the rest_2 and anxiety strategies, the baseline window is entirely disjoint from the windows used for evaluation, ensuring no overlap between normalization data and evaluated samples. In the rest_5 strategy, the last three minutes of the resting period contribute to both the normalization window and the evaluation set; this intentional overlap is the source of performance inflation that this study quantifies. Subject-level train/validation/test partitioning was then applied to the normalized feature set, maintaining complete separation between training and test subjects at the classification stage.

Continuous physiological recordings were segmented into fixed-duration temporal windows to generate discrete samples for supervised learning. To assess the impact of temporal resolution, we evaluated four window durations (10, 20, 30, and 60 s). The comprehensive analysis was focused on the extreme window lengths (10 and 60 s), representing different trade-offs between near real-time classification and feature stability, respectively. Intermediate durations were explored in one experimental condition. For 60-s windows, anxiety samples corresponded to individual video clips, while rest samples were extracted as non-overlapping one-minute segments from the final three minutes of resting period. For 10-s windows, each original 60-s clip was subdivided into six consecutive non-overlapping segments, increasing the number of samples six-fold while enabling evaluation of near-real-time anxiety detection capabilities. Table 4 summarizes the configuration of each task.

**Table 4:** Summary of experimental tasks with window size and sample configuration.

Task	Window size	Anxiety clips	Rest clips
Task 1	60 s	3 (clips 1–3)	3 (last 3 min)${}^{a}$
Task 2	60 s	8 (clips 1–8)	3+5 (augmented)${}^{b}$
Task 3	10 s	3 (clips 1–3)	3 (last 3 min)${}^{a}$
Task 4	10 s	8 (clips 1–8)	3+5 (augmented)${}^{b}$

${}^{a}$Rest clips correspond to the last three 60-s segments of the 5-min resting period.

${}^{b}$Rest clips augmented from 3 to 8 samples using augmentation strategy (see Section 2.6).

### 2.5 Machine learning framework

Nine machine learning algorithms were evaluated: *K-Nearest Neighbors (KNN)*, which classifies samples based on proximity to labeled training examples using distance metrics; *Support Vector Classifier (SVC)* (26), which identifies optimal hyperplanes that maximally separate classes in high-dimensional feature spaces; *Decision Tree*, which constructs hierarchical rule-based models through recursive binary splits; *Random Forest (RF)* (27), which implements ensembles of decision trees trained on random subsets and aggregates predictions through majority voting; *Neural Network (Multi-Layer Perceptron)*, which employs interconnected layers of neurons with non-linear activation functions trained through backpropagation; *Quadratic Discriminant Analysis (QDA)*, which models class distributions as multivariate Gaussians with distinct covariance matrices for each class; *Linear Discriminant Analysis (LDA)*, which assumes shared covariance structure and projects data onto lower-dimensional spaces that maximize class separation; *XGBoost* (28), which implements gradient boosting with regularization by sequentially training decision trees where each corrects errors from previous iterations; and *TabPFN* (29), which employs a pre-trained transformer architecture that performs in-context learning on small-to-medium tabular datasets.

We implemented subject-level data splitting. The 52 participants were divided into training (35 participants), validation (7 participants), and test (10 participants) sets, ensuring complete separation at the participant level. For each configuration, models were trained on the training set and evaluated on the validation set to identify optimal hyperparameters. The hyperparameter search spaces for all classifiers are reported in Supplementary Material. The best-performing configuration was then assessed on the held-out test set, which remained completely unseen during both training and hyperparameter tuning.

We created five independent train-validation-test splits by systematically shifting participant allocations in increments of 10 participants while maintaining the 35-7-10 ratio. Participant-level independence was maintained within each split. Performance metrics are reported throughout this work as means and standard deviations across the five splits.

Model performance was evaluated using **balanced accuracy** as the primary metric, defined for binary classification as the average of sensitivity and specificity (Equation 1):

$$\text{Balanced Accuracy}=\frac{\text{TPR}+\text{TNR}}{2}$$ (1)

where TPR denotes true positive rate (sensitivity) and TNR denotes true negative rate (specificity). Balanced accuracy was selected because it appropriately handles potentially imbalanced class distributions by weighting both classes equally (30), regardless of their relative frequencies in the dataset.

### 2.6 Data augmentation

To address inherent class imbalance in Tasks 2 and 4, where anxiety samples outnumber rest samples by 2.67:1, we evaluated four strategies for handling imbalanced training data. Three approaches aimed to balance the class distribution by augmenting the minority rest class, while the fourth maintained the original imbalance. *Simple oversampling* (31) randomly replicates existing rest samples until class balance is achieved, providing the most straightforward augmentation method. *Synthetic Minority Over-sampling Technique (SMOTE)* (32) generates synthetic samples in feature space through linear interpolation between minority class examples and their k-nearest neighbors, introducing diversity while maintaining feature space topology. *Noise injection* (31) adds controlled Gaussian perturbations to minority class samples, creating variations that may improve model robustness to measurement artifacts. imbalanced training preserves the original 2.67:1 class ratio without any augmentation, solely relying on balanced accuracy as the evaluation metric to ensure equal weighting of both classes despite their unequal representation.

### 2.7 Statistical analysis

Throughout Section 3, classification performance is reported as mean balanced accuracy (±SD) aggregated across specific combinations of splits, classifiers, and experimental factors. Because the same participant splits and classifiers are re-evaluated across multiple conditions (normalization methods, baseline strategies, window durations, augmentation strategies), the resulting balanced accuracy values are not statistically independent. All comparisons were consequently assessed using a repeated-measures nonparametric framework in which the unit of analysis is a matched block: a fixed combination of split and classifier, together with any additional factor held constant across the compared conditions. For the baseline-strategy comparison, each block corresponds to one split-classifier combination aggregated across normalization methods; for the normalization-method comparison, each block corresponds to one split-classifier combination aggregated across baseline strategies; for the window-duration comparison, each block corresponds to one split-classifier-baseline-strategy combination aggregated across the six full-feature normalization methods; for the augmentation-strategy comparison, blocks are defined analogously. For each analysis, an omnibus Friedman test (33) was performed first. Where significant, post-hoc comparisons used Wilcoxon signed-rank tests (34) with Bonferroni-Holm correction (35) for the baseline-strategy comparison (three levels), and Nemenyi tests (36, 37) for comparisons with more than three levels (eight normalization methods, four window durations, four augmentation strategies). For every pairwise comparison we additionally computed the mean paired difference in balanced accuracy with a 95% bootstrap confidence interval, the matched-pairs rank-biserial correlation as effect size, and Kendall’s W (38) for each omnibus effect. Full pairwise results, confidence intervals, effect sizes, and forest plots are reported in Supplementary Material, Section S2.

## 3 Results

### 3.1 Classification performance and statistical validation

Of the nine classifiers described in Section 2.5, results are reported in the main text for the four with the highest mean balanced accuracy (TabPFN, QDA, Random Forest, XGBoost, in decreasing order of mean performance in Task 1), consistently across baseline strategies and normalization methods; the remaining five classifiers (SVC, Neural Network, LDA, KNN, Decision Tree) did not reach comparable performance and are omitted here for readability. Complete results for all nine classifiers, for every task, are reported in Supplementary Material, Section S4. Table 5 presents performance metrics for the four top-performing classifiers across all experimental tasks and baseline configurations. Results represent the mean ± standard deviation of balanced accuracy averaged across the six full-feature normalization methods (subtraction, delta, relative_delta, logarithm, zscore, minmax). None and partial normalization are not included in the performance results. For this section Tasks 2 and 4 were evaluated without data augmentation.

**Table 5:** Classification performance across experimental tasks.

Task	Classifier	Bal. Acc. Rest_5	Bal. Acc. Rest_2	Bal. Acc. Anxiety
Task 1	TabPFN	0.92 ± 0.03	**0.85 ± 0.05**	**0.85 ± 0.05**
	XGBoost	0.89 ± 0.04	0.77 ± 0.04	0.81 ± 0.04
	RF	0.89 ± 0.04	0.80 ± 0.04	0.81 ± 0.06
	QDA	**0.93 ± 0.05**	0.84 ± 0.06	0.81 ± 0.07
Task 2	TabPFN	0.87 ± 0.05	0.75 ± 0.06	**0.76 ± 0.04**
	XGBoost	0.83 ± 0.04	0.72 ± 0.07	0.73 ± 0.05
	RF	0.82 ± 0.05	0.71 ± 0.07	0.70 ± 0.05
	QDA	**0.89 ± 0.04**	**0.81 ± 0.08**	0.72 ± 0.05
Task 3	TabPFN	0.78 ± 0.05	0.74 ± 0.08	**0.77 ± 0.06**
	XGBoost	**0.79 ± 0.04**	0.74 ± 0.06	**0.77 ± 0.05**
	RF	**0.79 ± 0.03**	**0.75 ± 0.05**	**0.77 ± 0.04**
	QDA	**0.79 ± 0.05**	0.73 ± 0.07	0.71 ± 0.05
Task 4	TabPFN	**0.77 ± 0.02**	**0.71 ± 0.05**	0.64 ± 0.04
	XGBoost	0.76 ± 0.03	0.70 ± 0.04	**0.66 ± 0.04**
	RF	0.74 ± 0.04	0.68 ± 0.04	0.65 ± 0.05
	QDA	**0.77 ± 0.04**	**0.71 ± 0.06**	0.62 ± 0.04

Mean balanced accuracy ± standard deviation aggregated across normalization methods. Best result per task and normalization in bold.

**Figure 3:**
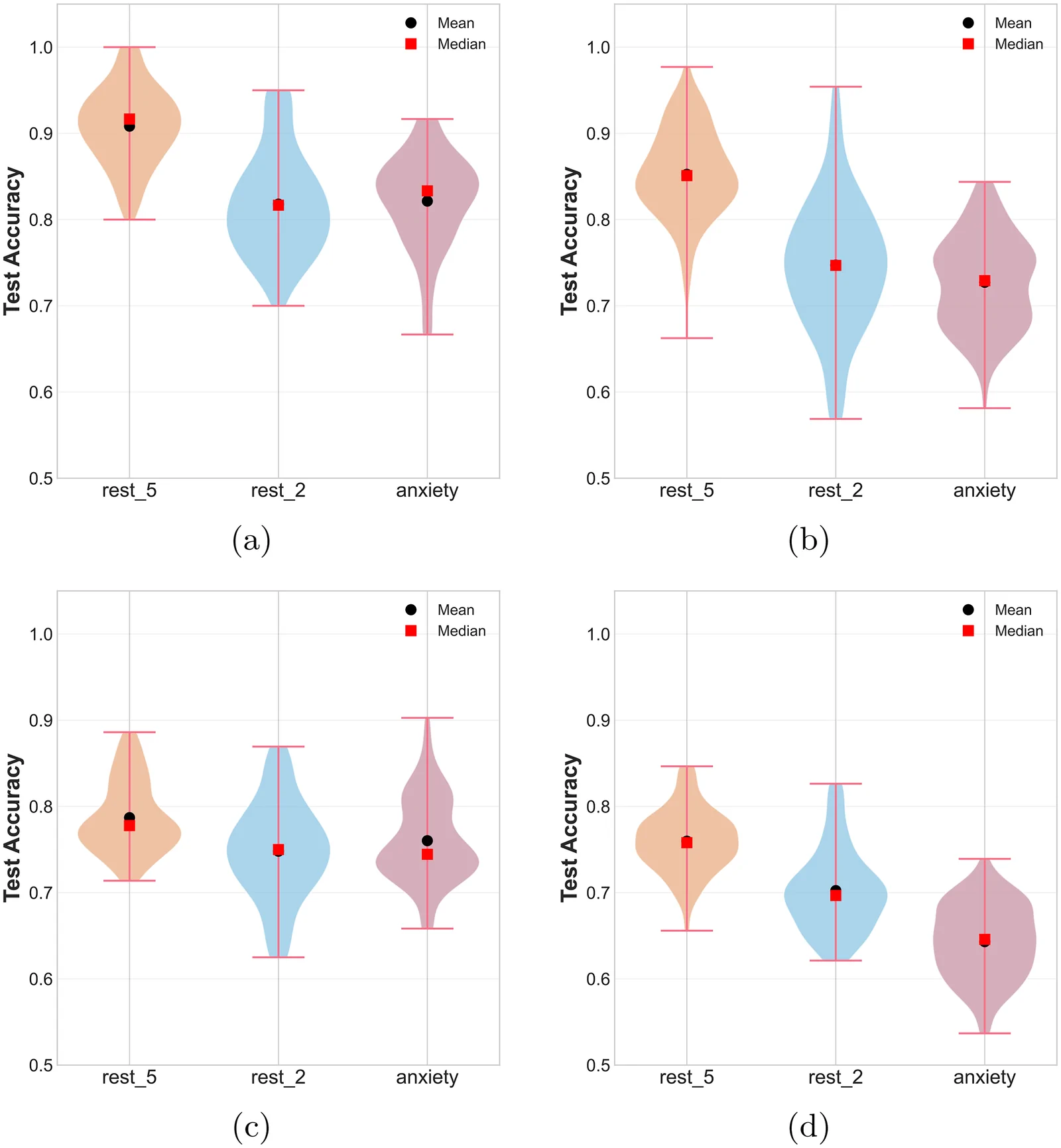
Balanced test accuracy distribution across baseline strategies for top four classifiers, aggregated over six full-feature normalization approaches. **(a)** Task 1: (3/3 clips, 60 s). **(b)** Task 2: (8/3 clips, 60 s). **(c)** Task 3: (3/3 clips, 10 s). **(d)** Task 4: (8/3 clips, 10 s). Violin plots display probability density distribution with median (red square), mean (black circle), and quartile ranges (horizontal red lines).

Classification performance varied with baseline strategy and task configuration, with no single classifier dominating across all conditions (mean balanced accuracy: TabPFN 0.78±0.05, XGBoost 0.76±0.05, RF 0.76±0.05, QDA 0.77±0.06). The rest_5 baseline consistently achieved the highest accuracies across all four tasks (Figure 3). In Task 1 (60 s, 3 clips), rest_5 reached average balanced accuracy 0.91, substantially outperforming both test-exclusive baseline normalization strategies (rest_2: 0.82, anxiety: 0.83). This performance gap persisted across all configurations: Task 2 (60 s, 8 clips) showed rest_5 at 0.86 vs. 0.75 (rest_2) and 0.73 (anxiety); Task 3 (10 s, 3 clips) exhibited rest_5 at 0.79 vs. 0.75 (rest_2) and 0.76 (anxiety); Task 4 (10 s, 8 clips) demonstrated rest_5 at 0.76 vs. 0.70 (rest_2) and 0.64 (anxiety).

Rest_5 consistently outperformed the test-exclusive baseline normalization by 0.03–0.13 mean balanced accuracy percentage points across all four experimental configurations. Anxiety and rest_2 baselines showed comparable performance across most tasks (differences <0.03 points), though their relative ordering varied slightly between configurations. Performance gaps between baselines remained stable across window sizes (60 s vs. 10 s) and balancing strategies.

**Figure 4:**
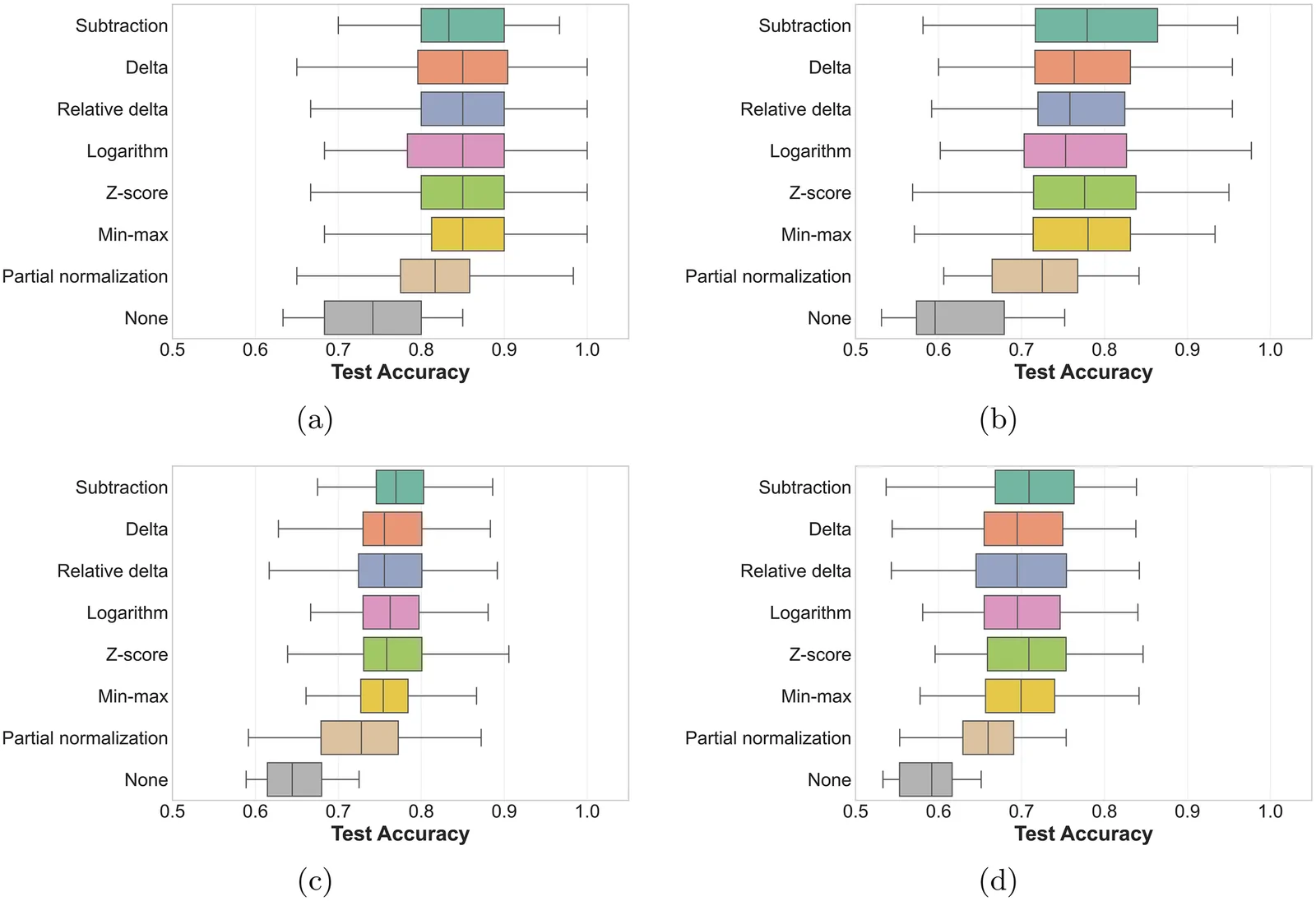
Distribution of balanced test accuracy across normalization methods for top four classifiers, aggregated over all three baseline strategies (rest_5, rest_2, anxiety). **(a)** Task 1 (3/3 clips, 60 s); **(b)** Task 2 (8/3 clips, 60 s); **(c)** Task 3 (3/3 clips, 10 s); **(d)** Task 4 (8/3 clips, 10 s). Boxplots display median (center line), interquartile range (box), and full range.

Figure 4 displays the distribution of test accuracies across normalization methods for the four experimental tasks, aggregated over all three baseline strategies and the top four classifiers. Across all four tasks, the six full-feature normalization approaches yielded similar performance distributions. Task 1 showed median accuracies clustering tightly around 0.85, with overlapping interquartile ranges spanning approximately 0.80-0.90. Task 2 exhibited slightly lower absolute performance levels, with median accuracies of 0.76–0.79 and interquartile ranges of 0.72–0.84. Task 3 demonstrated median accuracies of ≈0.76 with interquartile ranges of 0.73–0.80. Task 4 showed median accuracies of 0.69–0.71 and interquartile ranges of 0.66–0.76.

Two normalization approaches consistently deviated from the full-feature normalization approaches. No normalization produced substantially degraded performance across all tasks, with balanced accuracy decreasing by 0.11–0.18 percentage points. Partial normalization showed intermediate performance with median accuracies of 0.66–0.82 across tasks, falling consistently 0.03–0.05 percentage points below full-feature normalization.

**Figure 5:**
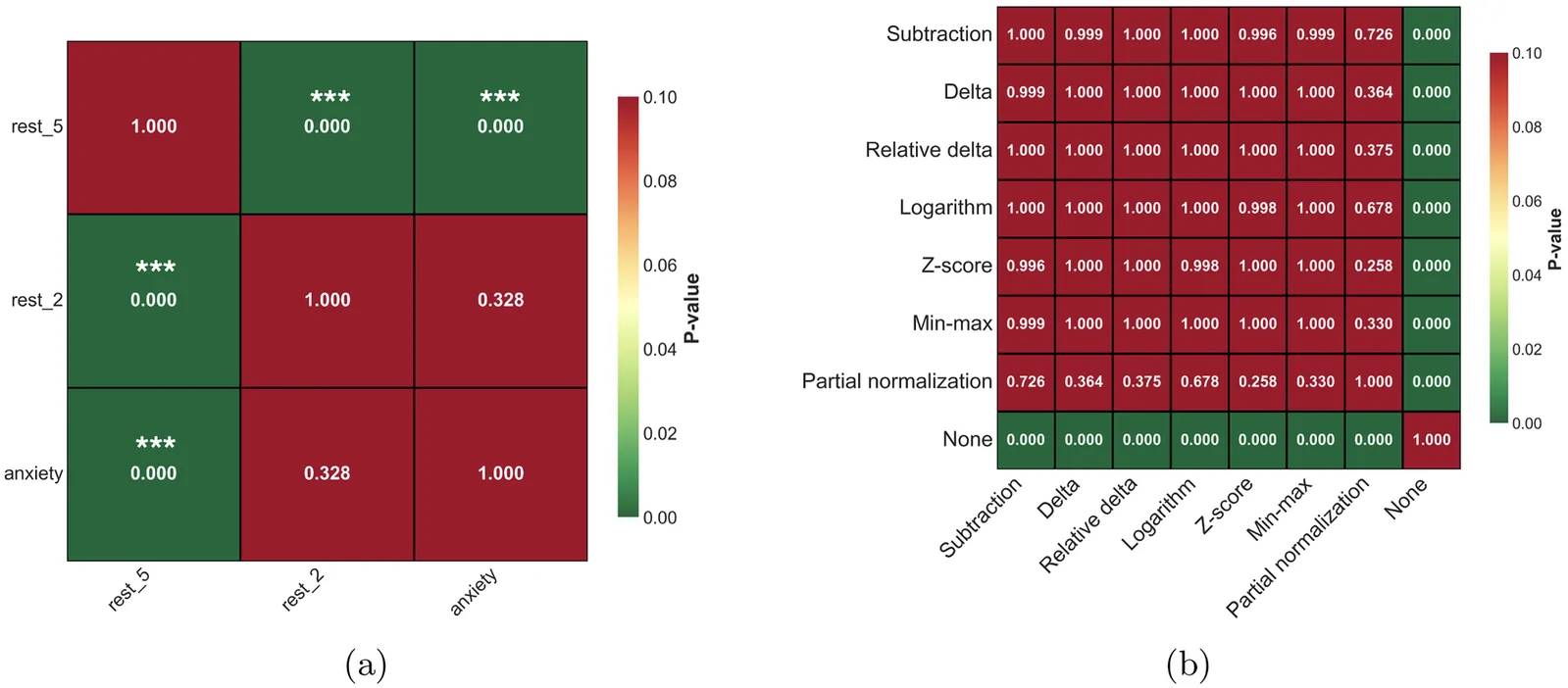
Statistical significance of performance differences for Task 1 across baseline strategies **(a)** and normalization methods **(b)**, based on Friedman tests with Wilcoxon signed-rank/Bonferroni-Holm **(a)** or Nemenyi **(b)** post-hoc comparisons. Each cell shows the corrected p-value for pairwise comparisons. Panel **(a)**: Baseline comparisons aggregated across all normalization methods and top four classifiers. Panel **(b)**: Normalization method comparisons aggregated across all baseline strategies and top four classifiers.

Statistical testing followed the framework described in Section 2.7. Figure 5 presents heatmaps of p-values for all pairwise comparisons, calculated using the top four classifiers across all baseline strategies and normalization methods. Color intensity reflects p-value magnitude: green indicates statistically significant differences (p<0.05), while red indicates non-significant differences (p≥0.05).

Panel (a) shows that rest_5 achieved significantly higher performance than both test-exclusive baseline normalization strategies (rest_5 vs. anxiety, p<0.001; rest_5 vs. rest_2, p<0.001). In contrast, no significant difference was observed between the two test-exclusive baseline normalization strategies (anxiety vs. rest_2, p=0.328). Panel (b) reveals a two-tier performance structure. No normalization (none) showed significantly lower performance compared to all other approaches, including partial normalization (p<0.001). Partial normalization showed consistently lower median accuracy than full-feature approaches, but this difference did not reach statistical significance after Nemenyi correction (p>0.05 for all comparisons). The six full-feature normalization methods formed a statistically homogeneous group with predominantly non-significant pairwise differences (p-values ranging from 0.996 to 1.000).

### 3.2 Window duration effects

To characterize how temporal window size affects anxiety detection balanced accuracy, we evaluated classification performance across four window durations: 10, 20, 30, and 60 s. This analysis included all nine classifiers evaluated in the study and was conducted exclusively on early-exposure task configuration (3 anxiety clips vs. 3 rest clips). Results were aggregated across all classifiers and full-feature normalization approaches, excluding none and partial normalization, and analyzed separately for (a) rest_5, (b) rest_2, and (c) anxiety baseline strategies.

**Figure 6:**
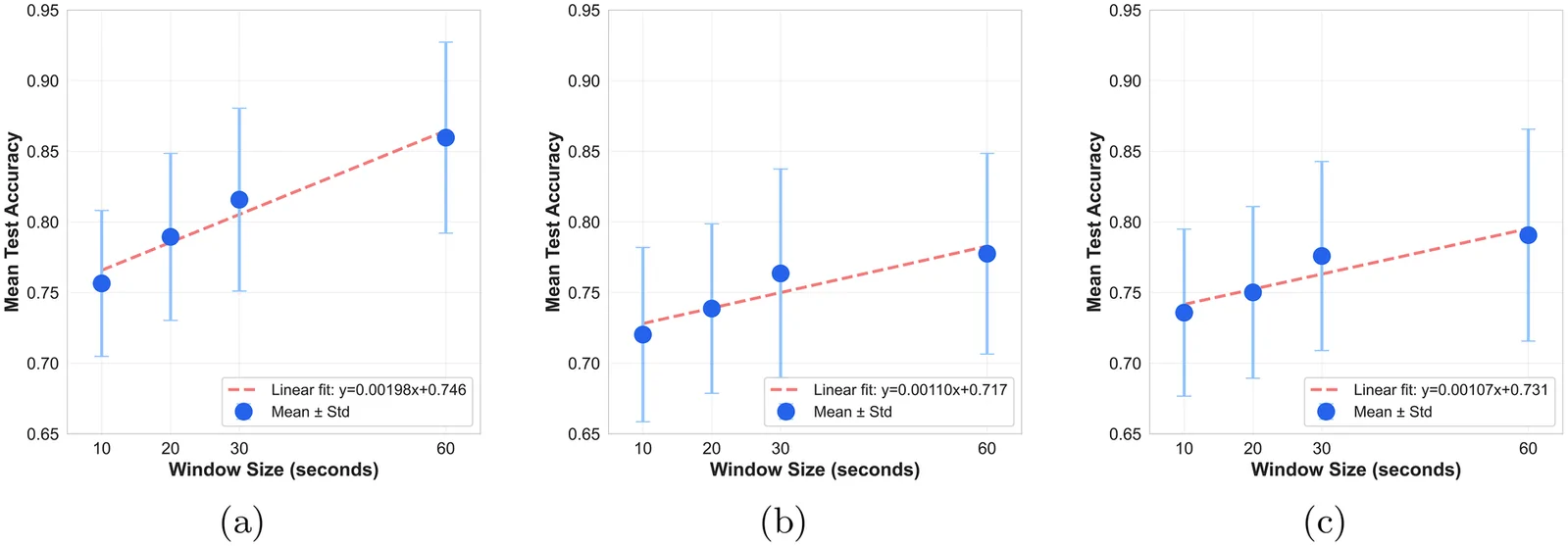
Classification performance as a function of temporal window size for early-exposure task configuration. Analysis includes all nine classifiers aggregated across full-feature normalization approaches. **(a)** Rest_5. **(b)** Rest_2. **(c)** Anxiety. Blue points represent mean balanced accuracy with error bars indicating standard deviation. Red dashed lines show linear regression fits.

Performance increased monotonically with window duration for baseline strategies (Figure 6). For rest_5, mean balanced accuracy increased from 0.76 (SD=0.05) at 10 s to 0.86 (SD=0.07) at 60 s, exhibiting a stronger linear trend ($R^{2}=0.9587$). For rest_2, mean balanced accuracy rose from 0.72 (SD=0.06) at 10 s to 0.78 (SD=0.07) at 60s, following a linear relationship ($R^{2}=0.8598$). For anxiety baseline balanced accuracy went from 0.74 (SD=0.06) for 10 s window to 0.79 (SD=0.08) for 60 s window with a linear relationship ($R^{2}=0.8779$). These trends were statistically confirmed by the repeated-measures framework described in Section 2.7 (Supplementary Material, Section S2.3, Figure S.5).

### 3.3 Impact of data augmentation strategies

**Figure 7:**
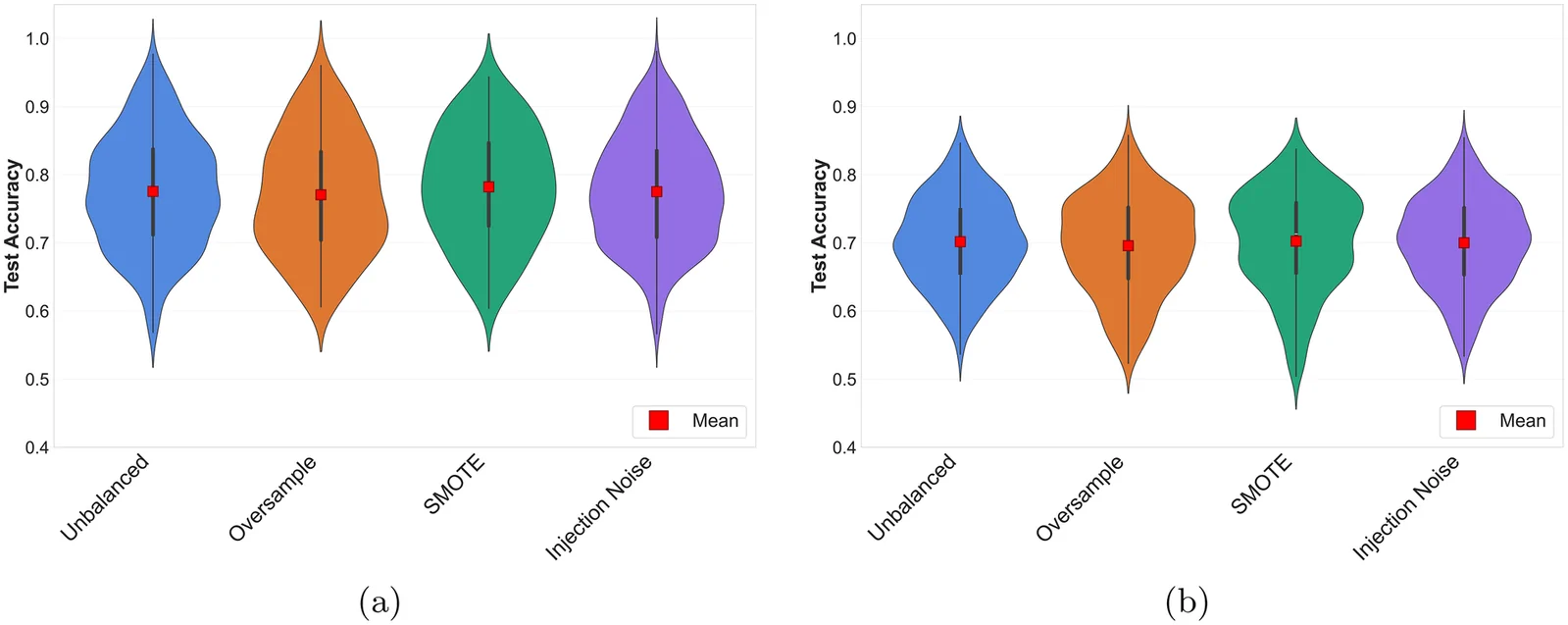
Distribution of balanced test accuracy across data augmentation strategies for top four classifiers, aggregated over all three baseline strategies (rest_5, rest_2, anxiety) and six full-feature normalization approaches. **(a)** Task 2: (8/3 clips, 60 s); **(b)** Task 4: (8/3 clips, 10 s). Red squares indicate mean balanced accuracy.

To assess the effectiveness of different augmentation strategies for addressing class imbalance, we compared four approaches across Tasks 2 and 4: imbalanced (no augmentation), oversample (simple duplication), SMOTE (synthetic minority oversampling technique), and injection noise (Gaussian noise addition). Figure 7 presents the distribution of test accuracies for each strategy, aggregated across the top four classifiers, all three baseline strategies, and the six full-feature normalization approaches. Table 6 summarizes mean balanced accuracies and standard deviations for each augmentation strategy.

**Table 6:** Mean balanced accuracy ± standard deviation aggregated across six full-feature normalization approaches, baselines, and the top 4 classifiers for the different augmentation strategies.

Task	Imbalanced	Oversample	SMOTE	Inj. noise
Task 2	0.78±0.08	0.77±0.09	0.78±0.08	0.77±0.08
Task 4	0.70±0.06	0.70±0.07	0.70±0.07	0.70±0.07

Task 2 (panel a) demonstrated consistent performance across all augmentation strategies. As shown in Table 6, mean accuracies ranged from 0.77±0.08 (oversample and injection noise) to 0.78±0.08 (imbalanced and SMOTE), representing differences of only 0.01 balanced accuracy points.

Task 4 (panel b) exhibited similar patterns of performance equivalence across augmentation strategies. All four approaches converged to identical mean accuracies of 0.70, with standard deviations ranging from 0.06 (imbalanced) to 0.07 (oversample, SMOTE, and injection noise), as reported in Table 6. This pattern of equivalence was statistically confirmed by the same framework (Section 2.7; Supplementary Material, Section S2.4, Figure S.6).

## 4 Discussion

This study investigated several fundamental methodological challenges in physiological anxiety detection: quantifying performance inflation effects from baseline normalization strategies, assessing habituation effects from repeated stimulus exposure, characterizing window duration impact on classification accuracy, and evaluating data augmentation approaches for imbalanced classes.

The most critical finding is that performance is artificially increased when the baseline period overlaps with the evaluation data. The rest_5 strategy, which computes normalization statistics from data that subsequently appears in test sets, consistently outperformed test-exclusive baseline normalization alternatives by 3–13 percentage points across all experimental configurations (Table 5, Figure 3). This advantage persisted regardless of window duration (60 s vs. 10 s), class balance (balanced vs. imbalanced), classifier complexity (QDA to TabPFN), or normalization method employed. Statistical testing confirmed highly significant differences between rest_5 and both test-exclusive baseline normalization (p<0.001, Figure 5a). Critically, rest_2 and anxiety baseline strategies demonstrated statistically equivalent performance despite employing fundamentally different physiological states for baseline computation. This equivalence rules out baseline physiological distribution as a driver of the performance gap. Baseline data quantity cannot be treated as an independent confound either: each subject contributes a fixed 5-min resting period, of which three minutes were consistently reserved for evaluation across all baseline strategies, leaving rest_5 and rest_2 structurally unequal in size within this dataset rather than independently adjustable. Furthermore, resting-state physiological features have been shown to stabilize within approximately two minutes, including in psychophysiological stress research (39, 40), indicating that the additional baseline data used by rest_5 would not be expected to substantially improve the accuracy of the normalization statistics. Temporal overlap is therefore the most plausible explanation for the observed advantage of rest_5 over both test-exclusive strategies, rather than differences in baseline content. The statistical equivalence of anxiety and rest baselines indicates that normalization is robust to baseline emotional state, enabling application-specific baseline protocol design. Across all tasks, these strategies achieved nearly identical accuracies, while rest_5 consistently exceeded both by substantial margins. When baseline measurements are included in the test set, models are evaluated on the same data used to define normalization parameters. This creates artificially predictable patterns where baseline samples cluster near reference values, inflating classification accuracy. The consistency of this effect across experimental conditions suggests that differences in baseline normalization and evaluation protocols may contribute to variation in reported accuracies across studies, highlighting the importance of transparent methodological reporting to enable fair performance comparisons and accurate assessment of clinical applicability. Rest_5 was included not as a candidate normalization strategy, but to quantify, within a controlled setting, the magnitude of performance inflation that may affect anxiety and related physiological classification studies reporting comparably high accuracies, where similar baseline-evaluation overlap could be present but is rarely reported explicitly. From a deployment perspective, rest_5 has no realistic prospective or retrospective counterpart: because its normalization statistics are computed from the same samples subsequently classified, replicating it on new, unseen recordings would require knowing in advance which windows constitute rest, the very outcome the system is intended to predict. Rest_2, in contrast, corresponds to a deployment-realistic scenario, in which a short baseline period precedes continuous monitoring, analogous to onboarding routines already used in consumer wearable devices. The duration of such a baseline window may vary depending on application requirements and desired personalization accuracy; importantly, including this window within the evaluation data, as illustrated by rest_5, introduces normalization leakage and can artificially inflate performance estimates, with the magnitude of this bias expected to increase as the baseline duration grows relative to the evaluation period. In practical deployment scenarios, where calibration may require approximately 10 to 30 min and predictions are generated over substantially longer periods (e.g., 24 h), the proportion of data directly affected by such overlap would be relatively small (approximately 0.7%–2%). Avoiding this form of leakage therefore remains methodologically important, but its impact on overall performance estimates is expected to be limited when the baseline period represents only a small fraction of the monitoring duration. Moreover, a short rest-based baseline of this kind is realistic for anxiety and stress detection in daily living, since individuals have the opportunity to record such periods under ordinary, low-arousal conditions. These considerations extend beyond controlled laboratory settings. In continuous, free-living monitoring, where a dedicated rest phase cannot be scheduled, the baseline should instead be identified post-hoc as the calmest naturally occurring period, recomputed separately for each monitoring session rather than fixed once for an individual, and excluded from model training and evaluation regardless of how it is defined (41). This suggests that the core requirement for unbiased normalization, disjointness between baseline and evaluation data, generalizes beyond controlled paradigms to real-world monitoring, even when the baseline itself must be defined post-hoc rather than prospectively. Beyond the primary baseline normalization effect, comparing tasks with different numbers of anxiety clips revealed evidence of habituation. Classification performance systematically decreased when using extended exposure tasks (e.g., Task 2: 0.71–0.81 with test-exclusive baseline normalization) compared to early exposure tasks (e.g., Task 1: 0.77–0.85), despite identical normalization strategies and window durations. This 4–7 percentage point reduction aligns with habituation effects documented in exposure therapy literature (17, 18), where repeated stimulus presentation progressively attenuates physiological responses, suggesting that cumulative exposure duration represents an important consideration in experimental design for physiological anxiety detection. This interpretation is confirmed by a direct quantification of physiological response magnitude across clips (Supplementary Material, Section S.6): heart rate elevation relative to each participant’s resting reference decreased significantly from early- to extended-exposure clips, consistent with cardiac habituation to repeated stimulus presentation. Respiration and EDA did not show a comparable, statistically significant attenuation.

Normalization method choice exhibited structured patterns across all experimental configurations. Six full-feature normalization approaches (subtraction, delta, relative_delta, logarithm, zscore, minmax) consistently formed a statistically homogeneous performance tier (Figure 4), with predominantly non-significant pairwise differences (p-values 0.996-1, Figure 5b) and median accuracies differing by less than 2–3 percentage points. This equivalence demonstrates that once feature normalization is applied, the specific mathematical transformation has minimal impact on classification performance, enabling method selection based on interpretability or computational efficiency rather than accuracy gains. In sharp contrast, complete absence of normalization degraded performance by 11–18 percentage points, while partial normalization (normalizing only three features as in (5)) showed intermediate deficits of 3–5 percentage points. This performance structure reveals two statistically distinct tiers, normalized vs. non-normalized approaches, with correction of inter-individual physiological variability being essential for unmasking anxiety-related patterns, whereas the choice among normalization methods, whether applied to the full feature set or only a subset, is largely interchangeable in terms of statistical performance.

Window duration analysis revealed trade-offs between near-real-time capability and classification accuracy. Evaluation-independent baseline normalization accuracies increased monotonically from 0.72–0.74 at 10 s to 0.78–0.79 at 60 s (Figure 6). This reflects fundamental constraints: short windows (10 s) contain only 10–15 cardiac cycles at normal resting heart rate (60–100 bpm) and only 1–4 respiratory cycles at normal breathing rate (6–24 breaths per minute) (5, 6), limiting reliability of heart rate and respiratory variability metrics requiring adequate inter-beat and inter-breath intervals, while longer windows improve statistical stability through averaging over more physiological cycles. Near-real-time anxiety monitoring systems requiring low latency (10 s) must accept 6–8 percentage point balanced accuracy penalties relative to 60-s windows, whereas 60-s window analysis can leverage longer windows for maximum accuracy. This latency-accuracy trade-off is a recognized design constraint in real-time physiological monitoring (42), and the quantitative characterization provided in Figure 6 offers a basis for selecting the operating point appropriate to a given clinical deployment scenario. Unlike applications such as arrhythmia detection, where delayed alerts carry immediate safety consequences, anxiety states typically evolve over tens of seconds to minutes, making low latency beneficial, e.g., for real-time biofeedback during exposure-based therapy, but not strictly critical. Window duration is therefore better framed as a tunable operating point than a fixed constraint. Statistical testing confirmed this pattern: the Friedman omnibus test was significant for all three baseline strategies, and Nemenyi post-hoc comparisons showed significant pairwise differences with large effect sizes for nearly all window-duration transitions. Performance differences between rest_5 and test-exclusive baseline normalization (rest_2, anxiety) remained consistent across window durations, demonstrating that baseline normalization approach influences results regardless of window size.

Data augmentation analysis revealed consistent performance across four strategies addressing the 2.67:1 class imbalance. For both 60-s windows (Task 2: 0.77--0.78±0.08) and 10-s windows (Task 4: 0.70±0.06--0.07), synthetic sample generation approaches (SMOTE, noise injection) provided no statistically advantage over oversampling or imbalanced training with balanced accuracy evaluation. Balanced accuracy provides a more reliable performance estimate than standard accuracy when class imbalance exists, as it prevents models from achieving artificially high scores by simply predicting the majority class. Artificial sample generation through duplication, interpolation, or noise injection did not improve upon natural variability in autonomic responses. This equivalence was confirmed statistically: the Friedman omnibus test was not significant for either task, and pairwise effect sizes among the four augmentation strategies were negligible, indicating that the observed differences reflect sampling variability rather than a genuine advantage of any augmentation strategy. Imbalanced training with balanced accuracy metrics is therefore preferable, avoiding computational overhead without performance loss. These conclusions apply to moderate imbalance ratios (2-3:1); extreme imbalance (10:1+) may benefit from synthetic augmentation or cost-sensitive learning.

Baseline normalization is widely employed in anxiety detection research to account for inter-individual physiological variability (4), yet methodological details, including baseline computation procedures, are mostly not fully documented (13, 14). Methodological variations in baseline handling may contribute to the range of performance metrics reported across studies. Test-exclusive baseline normalization approaches provide conservative estimates for clinical translation, which remain clinically meaningful for applications such as exposure therapy monitoring (5, 6) or workplace stress management (12) despite being lower than some previously reported accuracies of 90%–95% (5, 11).

The multi-domain structure of the feature set adopted here, combining central-tendency and dispersion statistics, temporal difference measures, and nonlinear Poincaré-based descriptors across ECG, RSP, and EDA, together with the leakage-aware handling of missing values and normalization statistics described in Sections 2.3, 2.4, aligns with recent wearable-based machine learning studies showing that both the choice of complementary feature categories and the preprocessing strategy substantially influence classification performance (43).

Despite these advances, our study is not without limitations. First, the sample comprised 52 participants recruited from a single center. To mitigate generalizability concerns, we implemented five independent subject-level train–validation–test splits across different data partitions. Second, data was acquired under controlled laboratory conditions using standardized video clips. Classification performance was lower in the extended-exposure condition, suggesting that cumulative exposure may reduce physiological discriminability, potentially due to habituation. In real-world settings, anxiety is often triggered by novel and unpredictable stressors, and additional confounds such as movement artifacts, spontaneous episodes, and variable baseline conditions would substantially alter the classification problem. It is therefore important to distinguish between the methodological conclusions of this work, regarding normalization leakage, habituation effects, and window duration trade-offs, which reflect general pipeline design choices applicable across wearable physiological monitoring studies, and the reported classification accuracies, which are specific to this controlled paradigm and should not be interpreted as indicative of real-world performance. To assess whether the normalization inflation effect generalizes beyond this primary dataset, the core pipeline was replicated on an independent dataset with different physiological modalities, population, and validation scheme (Supplementary Material, Section S.1). The replication confirmed the same directionality of results, supporting the interpretation that the observed inflation reflects a methodological consequence of test-inclusive baseline construction rather than a dataset-specific artifact. Third, we evaluated binary anxiety vs. rest classification. Clinical applications may require distinguishing anxiety severity levels or other affective states. Finally, anxiety labels were defined by experimental condition rather than concurrent validated clinical assessments. Although this approach is consistent with prior studies, it may not fully capture subjective anxiety experience or clinical symptom burden.

## 5 Conclusion

Our work showed that methodological choices substantially influence reported performance in physiological anxiety detection. Test-inclusive baseline normalization inflated balanced accuracy by 3–13 percentage points, a systematic bias that persisted across all classifiers, window sizes, and class balance conditions. Critically, the two test-exclusive strategies performed equivalently, confirming that temporal overlap rather than baseline selection drives the inflation. Extended stimulus exposure reduced performance by 4–7 percentage points under test-exclusive conditions, consistent with habituation-induced attenuation of physiological responses. Full-feature normalization improved balanced accuracy by 11–18 percentage points over no normalization, while the choice among full-feature methods had no statistically significant effect, enabling selection based on interpretability rather than accuracy. Window duration increased balanced accuracy from 72%–74% at 10 s to 78%–79% at 60 s, and data augmentation provided no measurable benefit under moderate class imbalance. Standardized reporting of baseline-test temporal boundaries and exposure-aware experimental designs are essential for reliable performance assessment in wearable affective monitoring. Although framed around anxiety detection, these findings and the evaluation framework may generalize to other autonomic-signal applications such as stress detection, emotion recognition, pain assessment, and cognitive-load estimation.

## Code availability

The code is available at https://github.com/alessandrotognotti/wearables_anxiety_classification.

## Data Availability

The dataset used in this study is publicly available on PhysioNet [20]: https://doi.org/10.13026/sq6q-zg04.
