# Supplementary material for: Baseline normalization choices inflate classification performance in wearable health monitoring: quantification and mitigation strategies
Source: Front Digit Health. 2026 Aug 18;8:1827279. doi: 10.3389/fdgth.2026.1827279 (PMC13529996; doi:10.3389/fdgth.2026.1827279)
Supplement: Supplementary file 1 [file Datasheet1.pdf]

SUPPLEMENTARY MATERIAL

S.1 Replication on an independent Dataset

To assess whether the normalization inflation effect is dataset-specific, the core pipeline was replicated on an independent publicly available dataset collected by Elgendi et al. (DOI: 10.3390/data7090132), comprising 19 participants with ECG and respiratory signals recorded at 500 Hz during exposure to alternating anxiety-inducing and non-anxiety-inducing video clips, without a dedicated resting phase. Given the structural differences with the primary dataset, several adaptations were necessary: EDA features were excluded due to signal unavailability; anxiety and rest class labels were derived from specific segments of the video sequence chosen to maximize physiological contrast while maintaining temporal separation from the normalization baseline; and a LOSO-CV scheme was adopted given the small sample size of 18 usable subjects. Only rest_2 and rest_5 baseline strategies were evaluated.
Figure S.1 displays the balanced accuracy distributions for rest_2 and rest_5. Rest_5 outperformed the test-exclusive rest_2 baseline (μ = 0.822 vs. μ = 0.769).

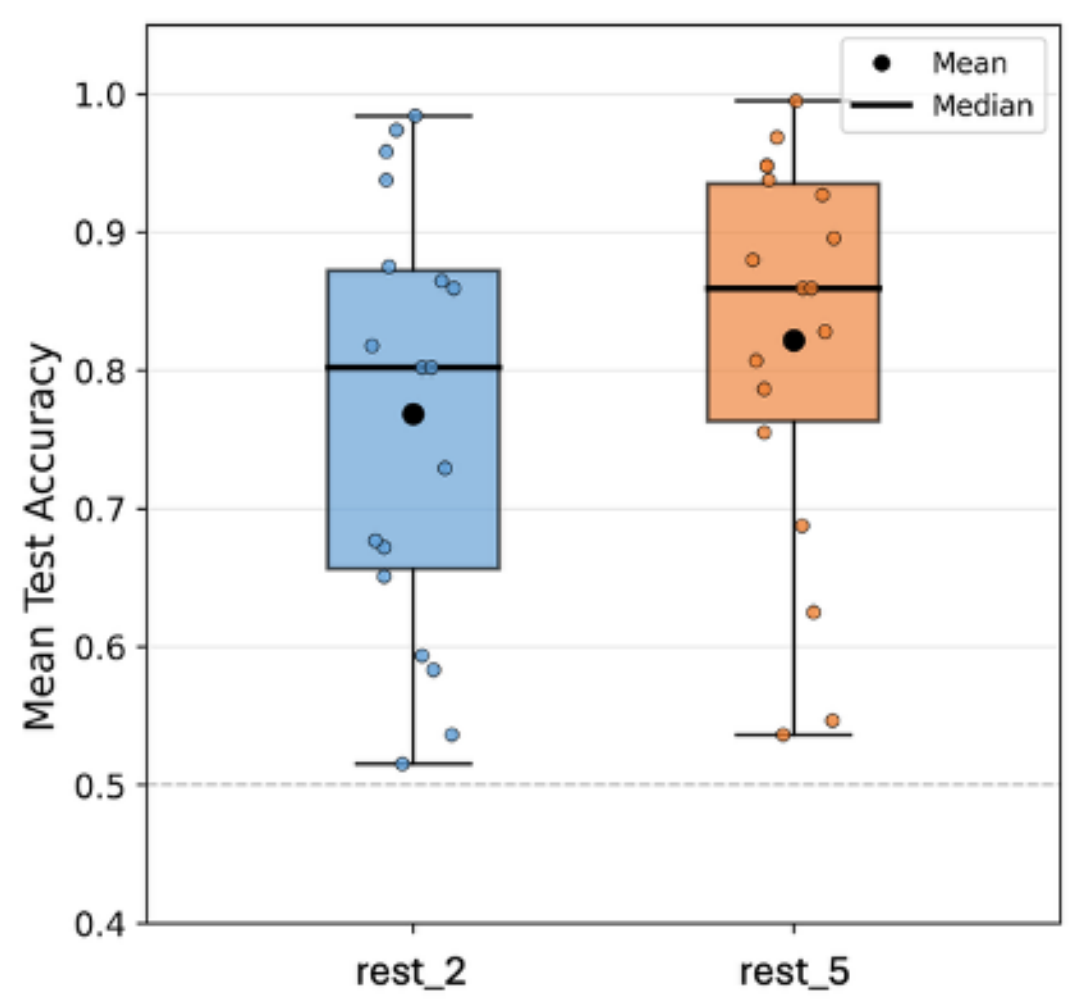

Figure S.1 : *Test accuracy distribution across baseline strategies for the top four classifiers on the Elgendi et al. dataset, aggregated over six full-feature normalization approaches using LOSO-CV (N=18). Each data point represents the mean balanced accuracy per subject.*

Figure S.2 displays the balanced accuracy distributions across normalization methods for both baseline strategies. The six full-feature normalization methods produced statistically equivalent performance under both rest_2 and rest_5, while the absence of normalization again yielded substantially lower performance in both conditions.

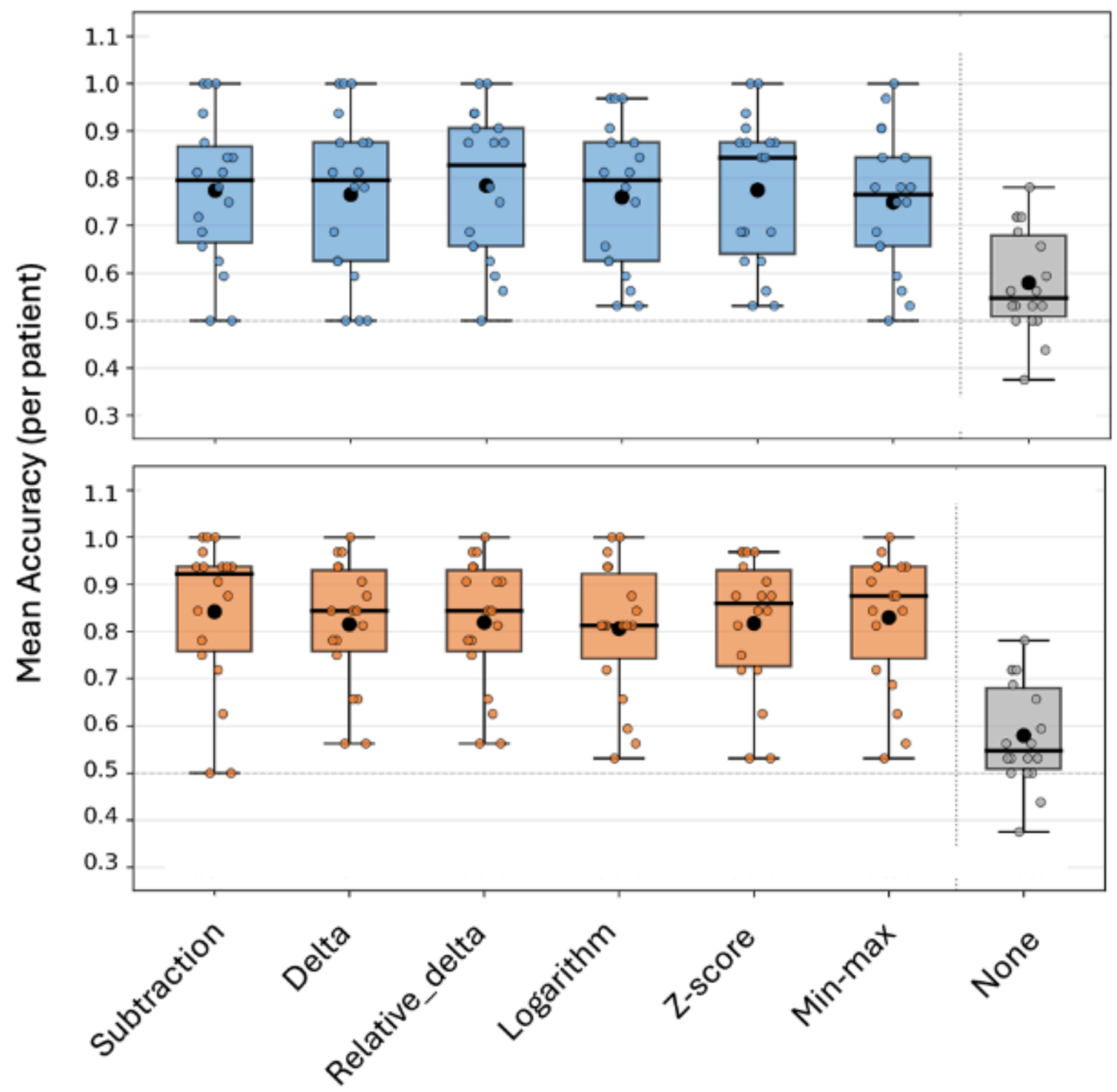

Figure S.2: *Mean balanced accuracy per subject across normalization methods for the Elgendi et al. dataset, shown separately for the test-exclusive baseline (rest_2, top, blue) and the test-inclusive baseline (rest_5, bottom, orange), aggregated over the top four classifiers using LOSO-CV (N=18). Each dot represents one subject.*

S.2. Effect Sizes and Confidence Intervals for Repeated-Measures Comparisons

To complement the significance testing described in Section 3.1, this section reports, for every pairwise comparison across the four analyses of this study, the mean difference in balanced accuracy with a 95% bootstrap confidence interval and the matched-pairs rank-biserial correlation r as a standardized effect size. Marker color in each figure indicates whether the comparison remained significant after the corrected post-hoc procedure (Wilcoxon signed-rank with Bonferroni-Holm for baseline and augmentation strategies, Nemenyi for normalization methods and window durations); a confidence interval excluding zero does not by itself imply significance under multiplicity correction, since the interval is not corrected for the number of simultaneous comparisons.

S2.1 Baseline strategies

Figure S.3 reports mean differences, 95% confidence intervals, and effect sizes for the three pairwise comparisons among baseline strategies (rest_5, rest_2, anxiety), for Task 1.

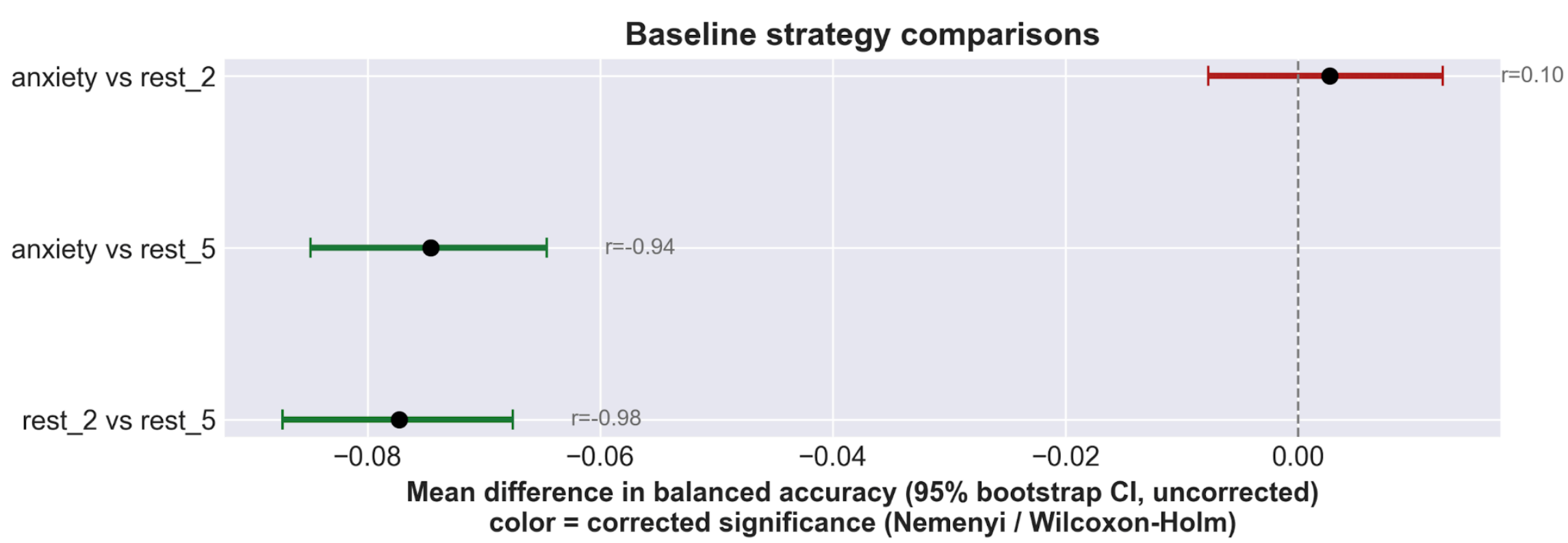

S2.2 Normalization methods

Figure S.4 reports the same statistics for all 28 pairwise comparisons among the eight normalization methods, for Task 1.

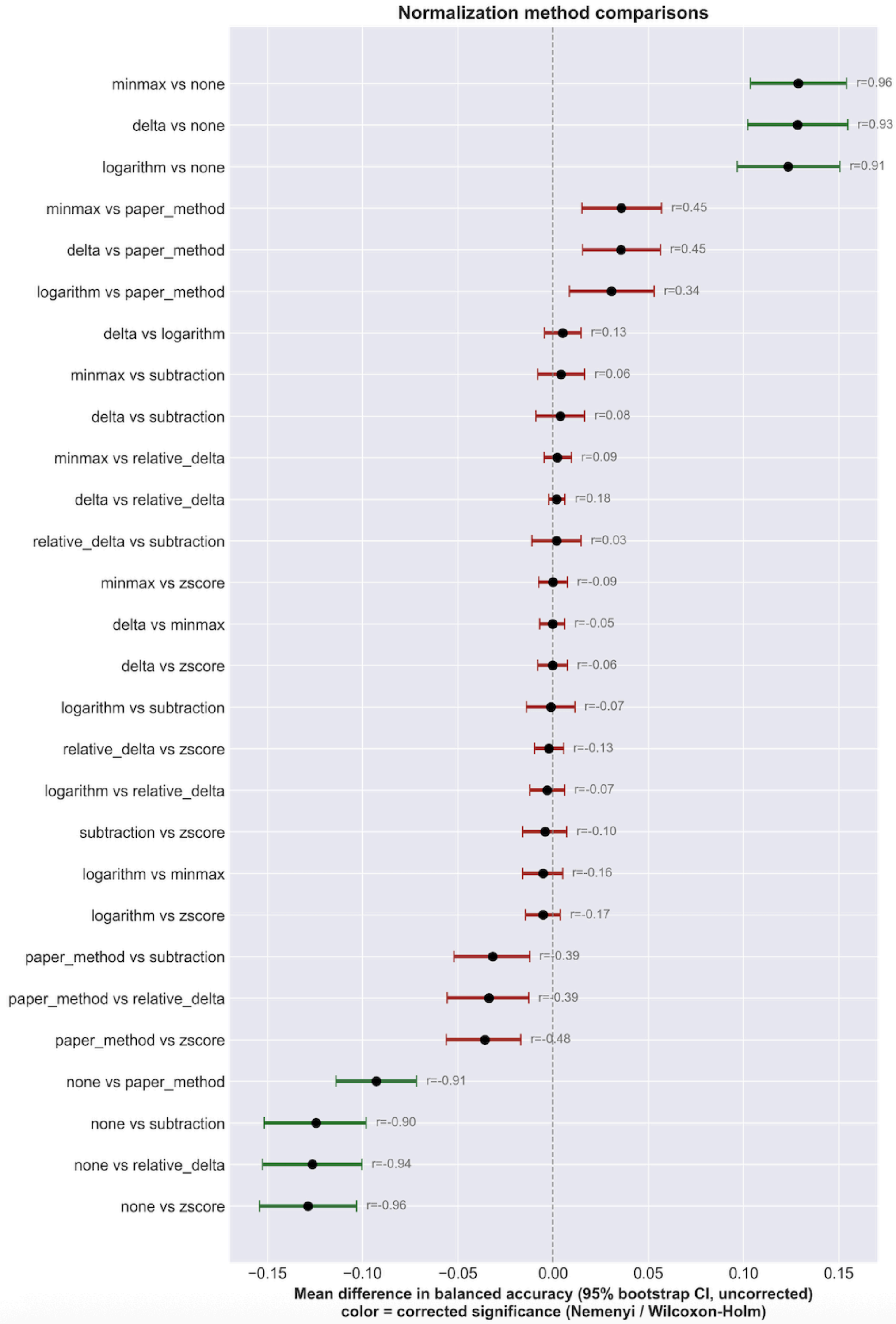

Figure S.4

S2.3 Window duration

Figure S.5 reports mean differences, confidence intervals, and effect sizes for all six pairwise comparisons among window durations (10s, 20s, 30s, 60s), for each of the three baseline strategies (rest_5, rest_2,anxiety), aggregated across all nine classifiers as in Figure 6.

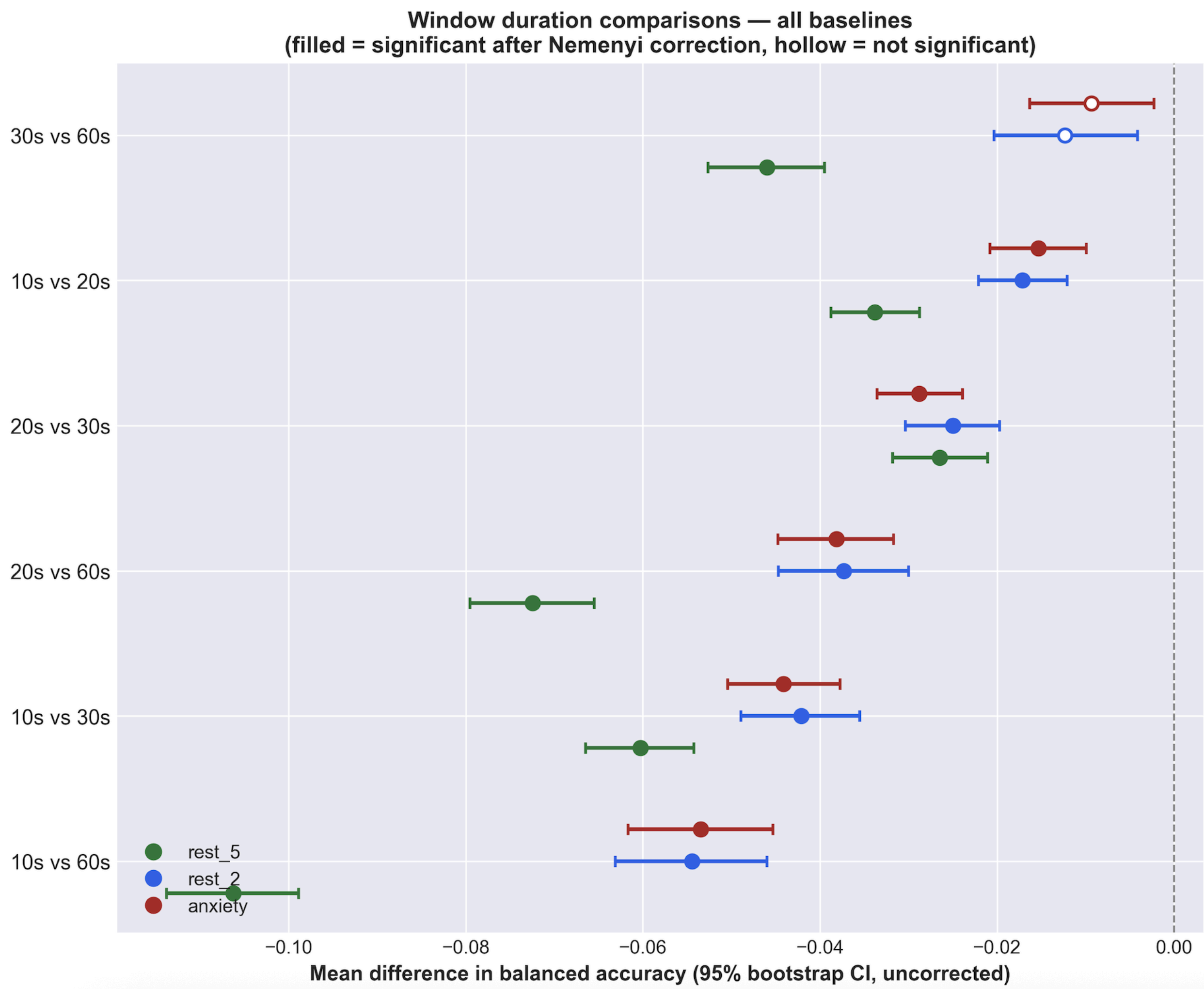

Figure S.5

S2.4 Augmentation strategies

Figure S.6 reports the same statistics for all six pairwise comparisons among the four augmentation strategies (Unbalanced, Oversample, SMOTE, Injection Noise), for Tasks 2, aggregated across the top four classifiers as in Figure 7.

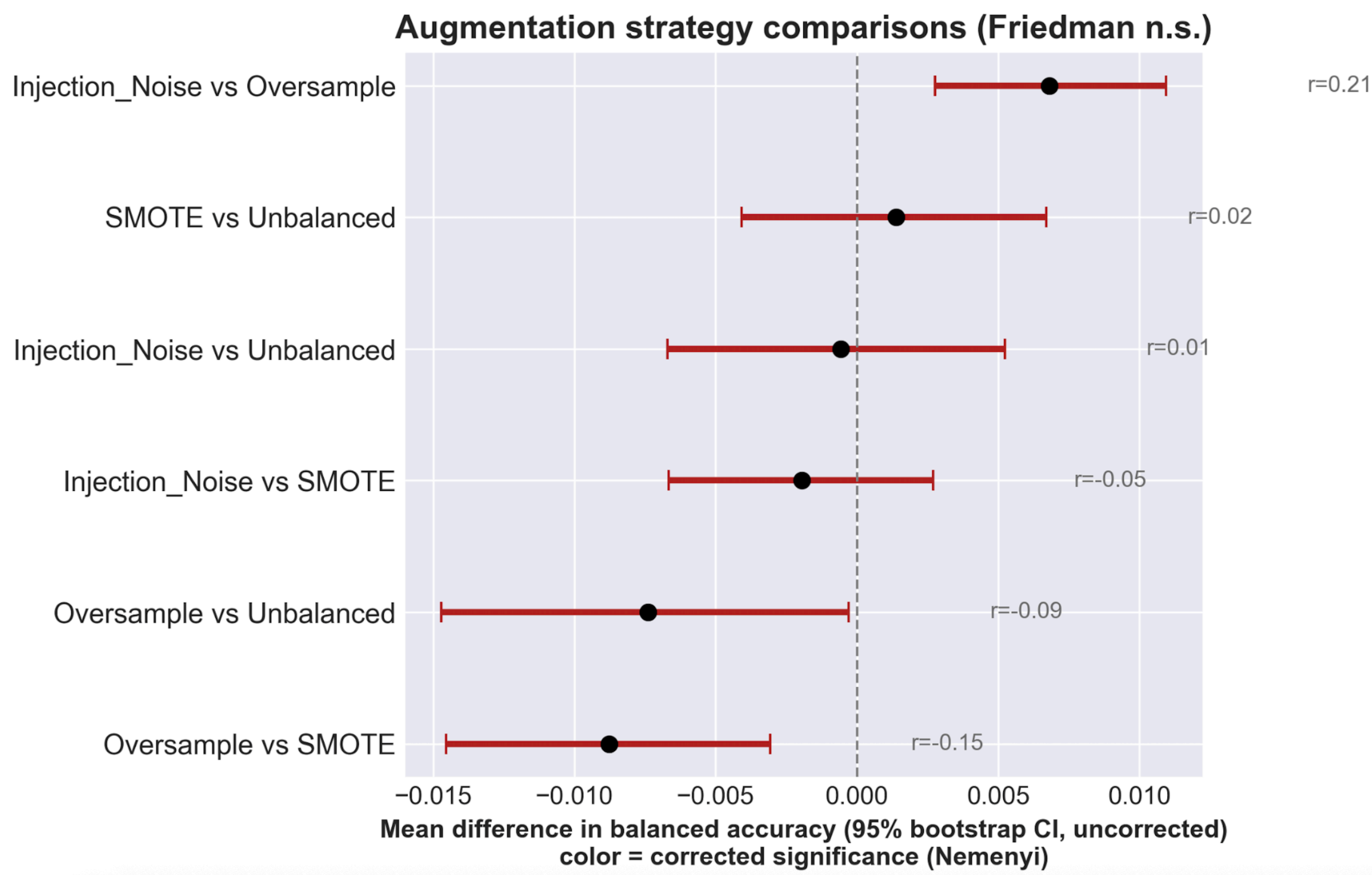

S.3 Statistical Results (for Task 2, 3, 4)

TASK 2

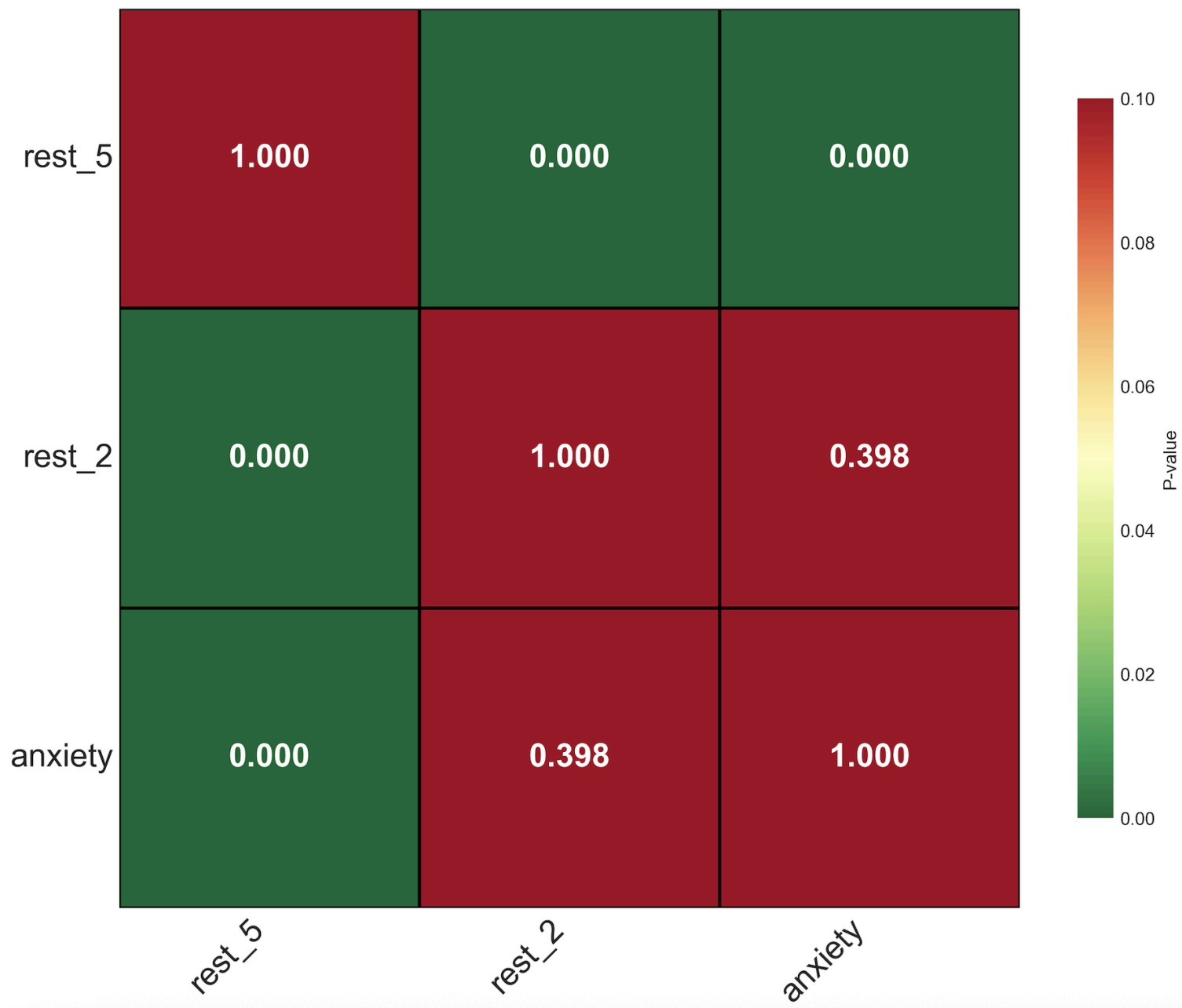

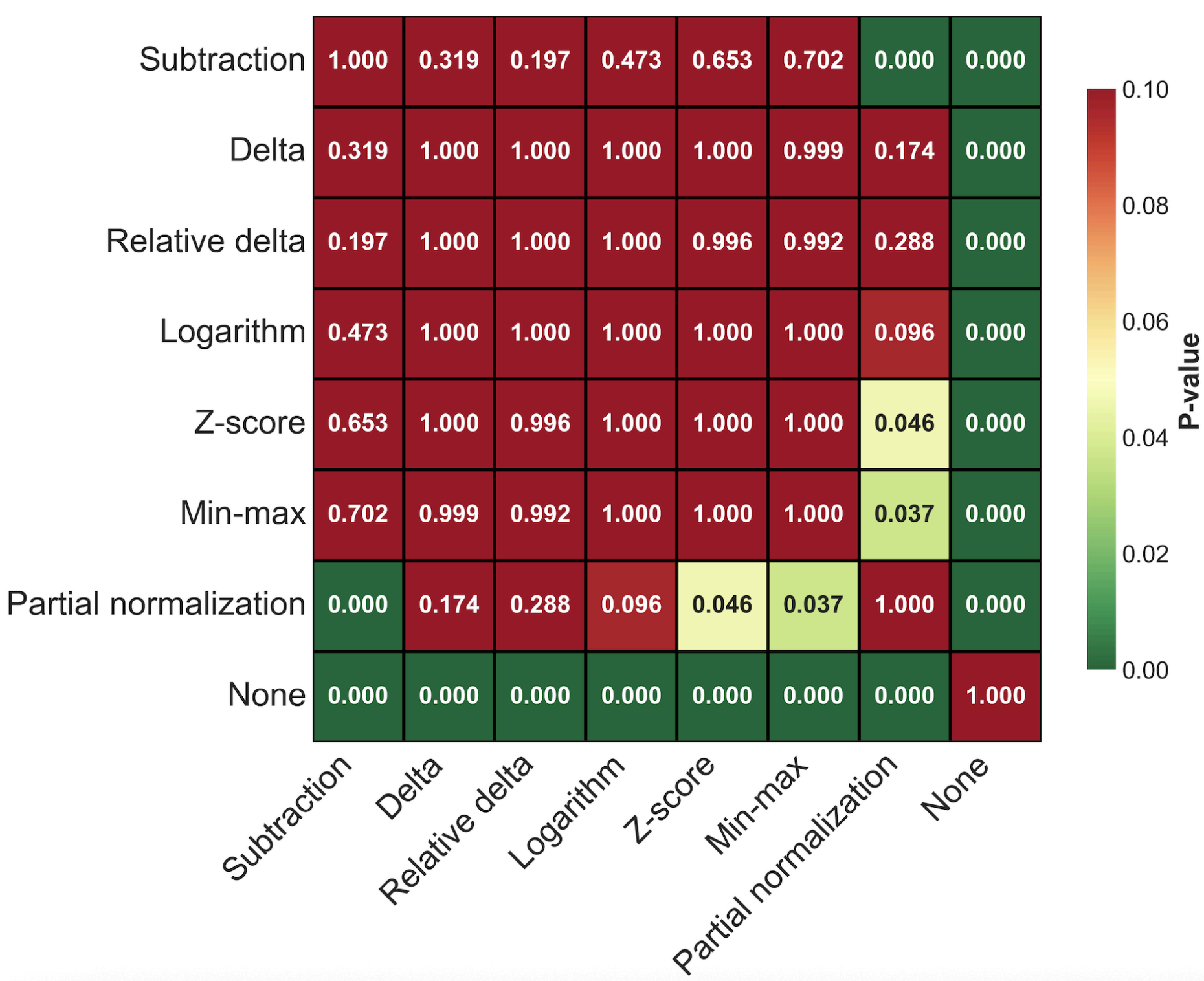

TASK 3

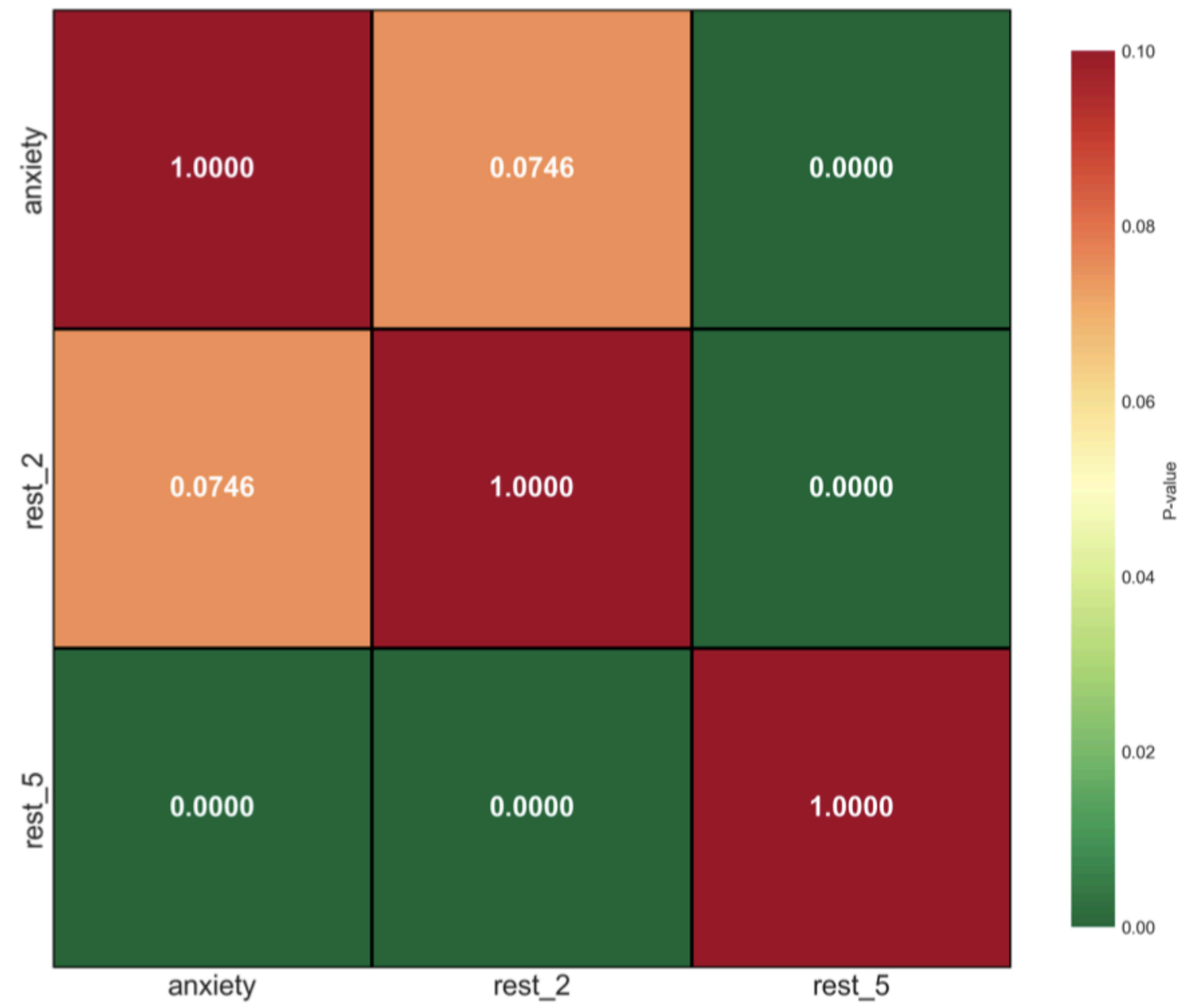

anxiety
rest_2
rest_5
1.0000
0.0746
0.0000
0.0746
1.0000
0.0000
0.0000
0.0000
1.0000
anxiety
rest_2
rest_5
0.10
0.08
0.06
0.04
0.02
0.00
P-value

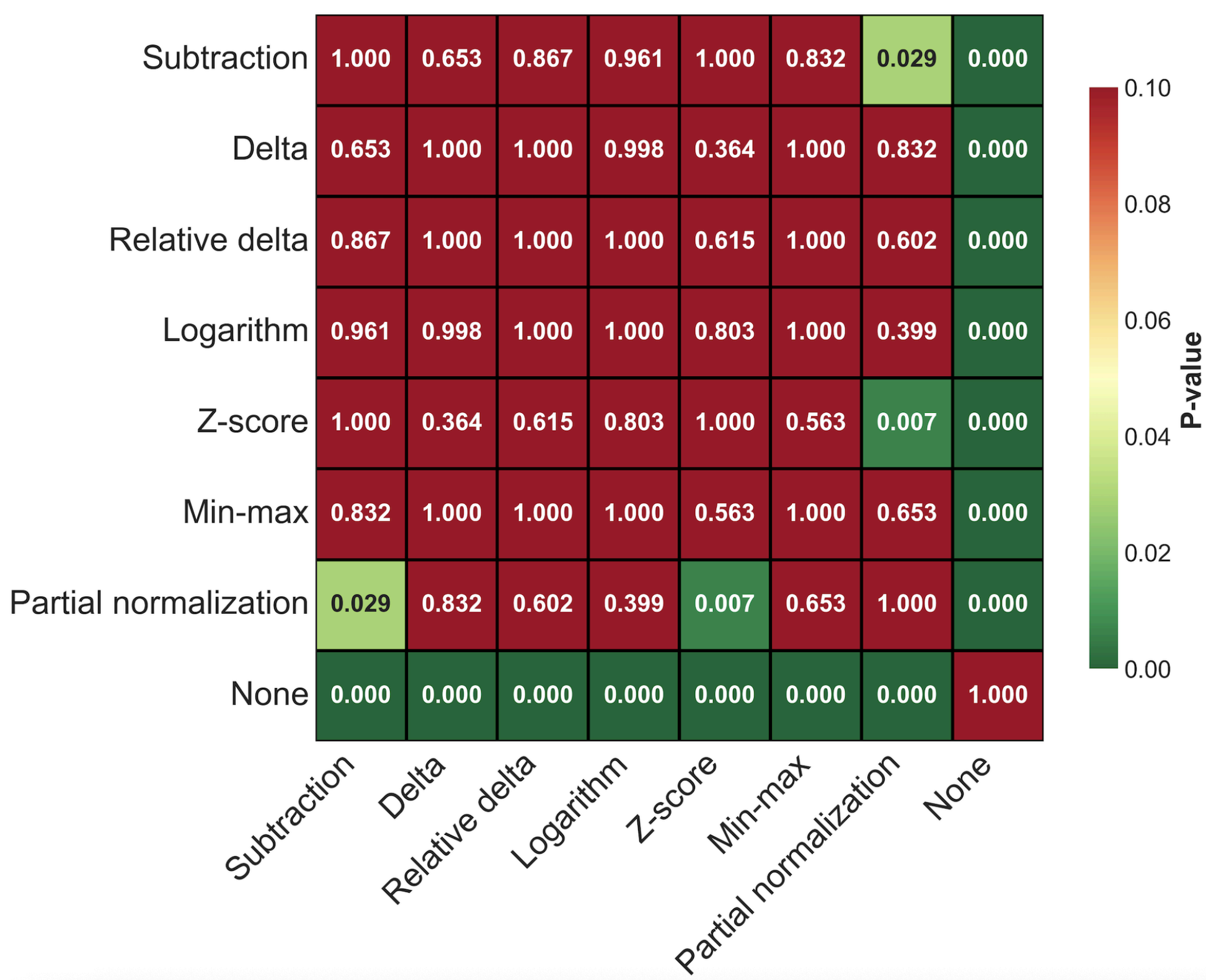

Subtraction
1.000 0.653 0.867 0.961 1.000 0.832 0.029 0.000
Delta
0.653 1.000 1.000 0.998 0.364 1.000 0.832 0.000
Relative delta
0.867 1.000 1.000 1.000 0.615 1.000 0.602 0.000
Logarithm
0.961 0.998 1.000 1.000 0.803 1.000 0.399 0.000
Z-score
1.000 0.364 0.615 0.803 1.000 0.563 0.007 0.000
Min-max
0.832 1.000 1.000 1.000 0.563 1.000 0.653 0.000
Partial normalization
0.029 0.832 0.602 0.399 0.007 0.653 1.000 0.000
None
0.000 0.000 0.000 0.000 0.000 0.000 0.000 1.000
Subtraction
Delta
Relative delta
Logarithm
Z-score
Min-max
Partial normalization
None
0.10
0.08
0.06
0.04
0.02
0.00
P-value

TASK 4

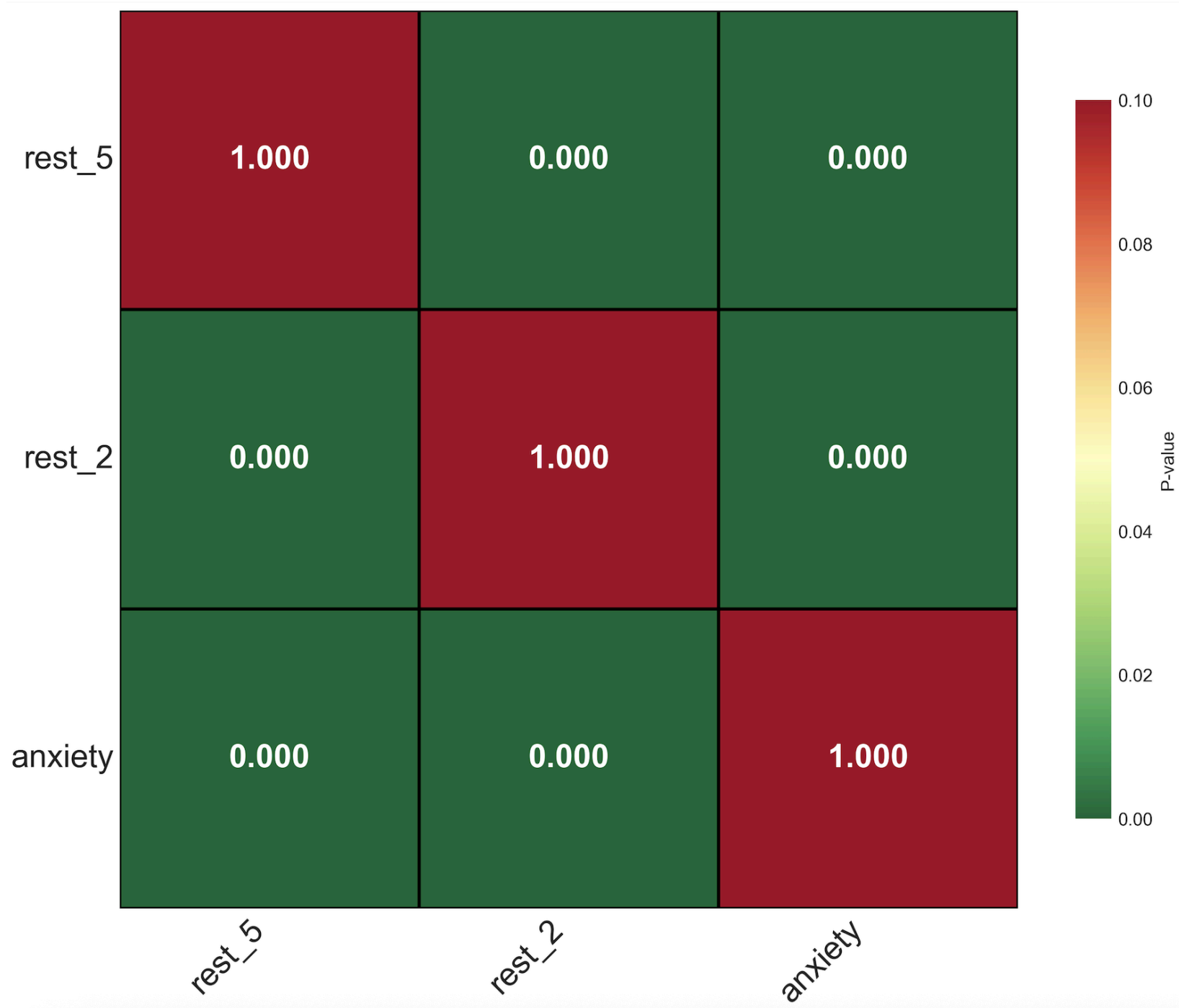
rest_5
rest_2
anxiety
1.000
0.000
0.000
0.000
1.000
0.000
0.000
0.000
1.000
rest_5
rest_2
anxiety
0.10
0.08
0.06
0.04
0.02
0.00
P-value

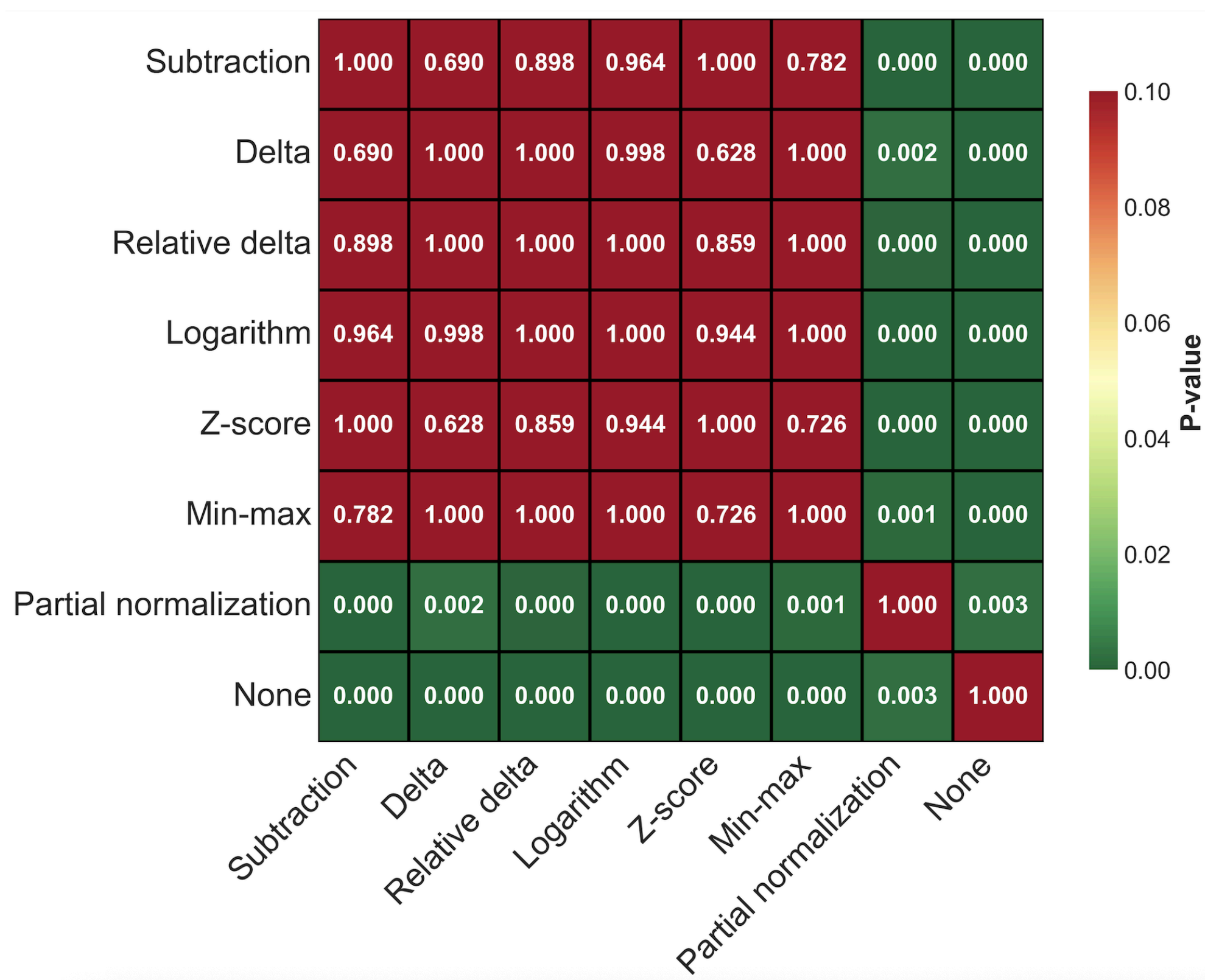
Subtraction
Delta
Relative delta
Logarithm
Z-score
Min-max
Partial normalization
None
1.000 0.690 0.898 0.964 1.000 0.782 0.000 0.000
0.690 1.000 1.000 0.998 0.628 1.000 0.002 0.000
0.898 1.000 1.000 1.000 0.859 1.000 0.000 0.000
0.964 0.998 1.000 1.000 0.944 1.000 0.000 0.000
1.000 0.628 0.859 0.944 1.000 0.726 0.000 0.000
0.782 1.000 1.000 1.000 0.726 1.000 0.001 0.000
0.000 0.002 0.000 0.000 0.000 0.001 1.000 0.003
0.000 0.000 0.000 0.000 0.000 0.000 0.003 1.000
Subtraction
Delta
Relative delta
Logarithm
Z-score
Min-max
Partial normalization
None
0.10
0.08
0.06
0.04
0.02
0.00
P-value

S4. Classification Results of All Tested Models

Section 3.1 reports results in the main text for the four classifiers with the highest mean balanced accuracy (TabPFN, QDA, Random Forest, XGBoost). Tables S.1-S.4 report the complete results for all nine classifiers evaluated in this study (KNN, SVC, Decision Tree, Random Forest, Neural Network, QDA, LDA, XGBoost, TabPFN), for each of the four experimental tasks, aggregated across the six full-feature normalization methods. Classifiers are ordered by decreasing mean balanced accuracy within each task. Task 2 and Task 4 reported results for the imbalanced configuration

task_1

Classifier	Bal. Acc. Rest_5	Bal. Acc. Rest_2	Bal. Acc. Anxiety
**TabPFN**	0.92 ± 0.03	0.85 ± 0.05	0.85 ± 0.05
**QDA**	0.93 ± 0.05	0.84 ± 0.06	0.81 ± 0.07
**RF**	0.89 ± 0.04	0.80 ± 0.04	0.81 ± 0.06
**XGBoost**	0.89 ± 0.04	0.77 ± 0.04	0.81 ± 0.04
**SVC**	0.86 ± 0.06	0.79 ± 0.07	0.80 ± 0.07
**Neural Network**	0.86 ± 0.05	0.75 ± 0.06	0.80 ± 0.07
**LDA**	0.81 ± 0.04	0.74 ± 0.06	0.82 ± 0.06
**KNN**	0.82 ± 0.07	0.73 ± 0.05	0.74 ± 0.07
**Decision Tree**	0.81 ± 0.05	0.73 ± 0.06	0.71 ± 0.08

task_2

Classifier	Bal. Acc. Rest_5	Bal. Acc. Rest_2	Bal. Acc. Anxiety
**QDA**	0.89 ± 0.04	0.81 ± 0.08	0.72 ± 0.05
**TabPFN**	0.87 ± 0.05	0.75 ± 0.06	0.76 ± 0.04
**XGBoost**	0.83 ± 0.04	0.72 ± 0.07	0.73 ± 0.05
**RF**	0.82 ± 0.05	0.71 ± 0.07	0.70 ± 0.05
**Neural Network**	0.78 ± 0.06	0.70 ± 0.07	0.70 ± 0.06
**SVC**	0.77 ± 0.07	0.66 ± 0.06	0.71 ± 0.07
**KNN**	0.81 ± 0.05	0.69 ± 0.07	0.62 ± 0.03
**Decision Tree**	0.74 ± 0.04	0.66 ± 0.06	0.66 ± 0.04
**LDA**	0.62 ± 0.04	0.61 ± 0.04	0.70 ± 0.07

task_3

Classifier	Bal. Acc. Rest_5	Bal. Acc. Rest_2	Bal. Acc. Anxiety
**RF**	0.79 ± 0.03	0.75 ± 0.05	0.77 ± 0.04
**XGBoost**	0.79 ± 0.04	0.74 ± 0.06	0.77 ± 0.05
**TabPFN**	0.78 ± 0.05	0.74 ± 0.08	0.77 ± 0.06
**QDA**	0.79 ± 0.05	0.73 ± 0.07	0.71 ± 0.05
**SVC**	0.76 ± 0.04	0.74 ± 0.05	0.73 ± 0.05
**Neural Network**	0.76 ± 0.05	0.73 ± 0.07	0.74 ± 0.07
**LDA**	0.72 ± 0.04	0.72 ± 0.04	0.74 ± 0.06
**Decision Tree**	0.74 ± 0.03	0.69 ± 0.06	0.72 ± 0.05
**KNN**	0.72 ± 0.04	0.69 ± 0.04	0.71 ± 0.05

task_4

Classifier	Bal. Acc. Rest_5	Bal. Acc. Rest_2	Bal. Acc. Anxiety
**TabPFN**	0.77 ± 0.02	0.71 ± 0.05	0.64 ± 0.04
**XGBoost**	0.76 ± 0.03	0.70 ± 0.04	0.66 ± 0.04
**QDA**	0.77 ± 0.04	0.71 ± 0.06	0.62 ± 0.04
**RF**	0.74 ± 0.04	0.68 ± 0.04	0.65 ± 0.05
**Neural Network**	0.72 ± 0.04	0.69 ± 0.05	0.65 ± 0.05
**SVC**	0.71 ± 0.04	0.66 ± 0.03	0.64 ± 0.05
**KNN**	0.71 ± 0.04	0.66 ± 0.03	0.60 ± 0.04
**Decision Tree**	0.69 ± 0.06	0.64 ± 0.05	0.62 ± 0.04
**LDA**	0.59 ± 0.03	0.57 ± 0.03	0.60 ± 0.04

S.5 Hyperparameters of Machine Learning Models Used

We report in this section the hyperparameter Table for each classifier.

Model	Hyperparameters
**k-Nearest Neighbors**	n neighbors ∈ {5, 10, 15}<br>weights ∈ {uniform, distance}
**Support Vector Classifier**	C ∈ {0.25, 0.5, 1}<br>kernel ∈ {linear, poly, rbf}
**Decision Tree**	max_depth ∈ {None, 5, 10, 15, 25, 50}<br>min_samples_split ∈ {2, 3, 5}
**Random Forest**	n_estimators ∈ {50, 100, 150, 250, 500, 1000, 1500}<br>max_depth ∈ {None, 10, 20, 30, 50, 100}<br>min_samples_split ∈ {2, 3, 5, 10}
**Neural Network**	hidden layer sizes ∈ {(100, 10), (50, 100, 50, 10),<br>(25, 50, 25, 10), (50, 100, 50, 10),<br>(25, 50, 100, 100, 50, 25, 10)}<br>early stopping ∈ {True, False}
**QDA**	default
**LDA**	default
**XGBoost**	n_estimators ∈ {100, 250, 500}<br>max_depth ∈ {3, 6, 10}<br>learning_rate ∈ {0.05, 0.1, 0.2}<br>subsample ∈ {0.8, 1.0}<br>colsample_bytree ∈ {0.8, 1.0}
**TabPFN**	default

Table S.5: Hyperparameter search spaces for each classifier

S.6 Physiological Response Magnitude Across Anxiety Clips

To support the interpretation of habituation discussed in Section 4, we quantified, for each of the 52 participants, the deviation of ECG, respiration, and EDA features from that participant's own resting-state reference (mean of the last three rest minutes) for each of the eight anxiety clips (Task 2 configuration), and tested for a trend across clip presentation order and a difference between early-exposure (clips 1-3) and extended-exposure (clips 4-8) responses. For each feature, *mean ρ* is the average per-participant Spearman correlation between clip presentation order and response magnitude, and *p_trend* is the p-value of a one-sample Wilcoxon test assessing whether this correlation differs from zero across participants. *Mean early* and *mean extended* are the average response magnitude over clips 1-3 and clips 4-8, respectively; *mean diff* is their difference; and *p_early_vs_extended* is the p-value of a paired Wilcoxon signed-rank test comparing the two, matched by participant. Table S.6 reports the results; Figure S.6 shows the cardiac response trend.

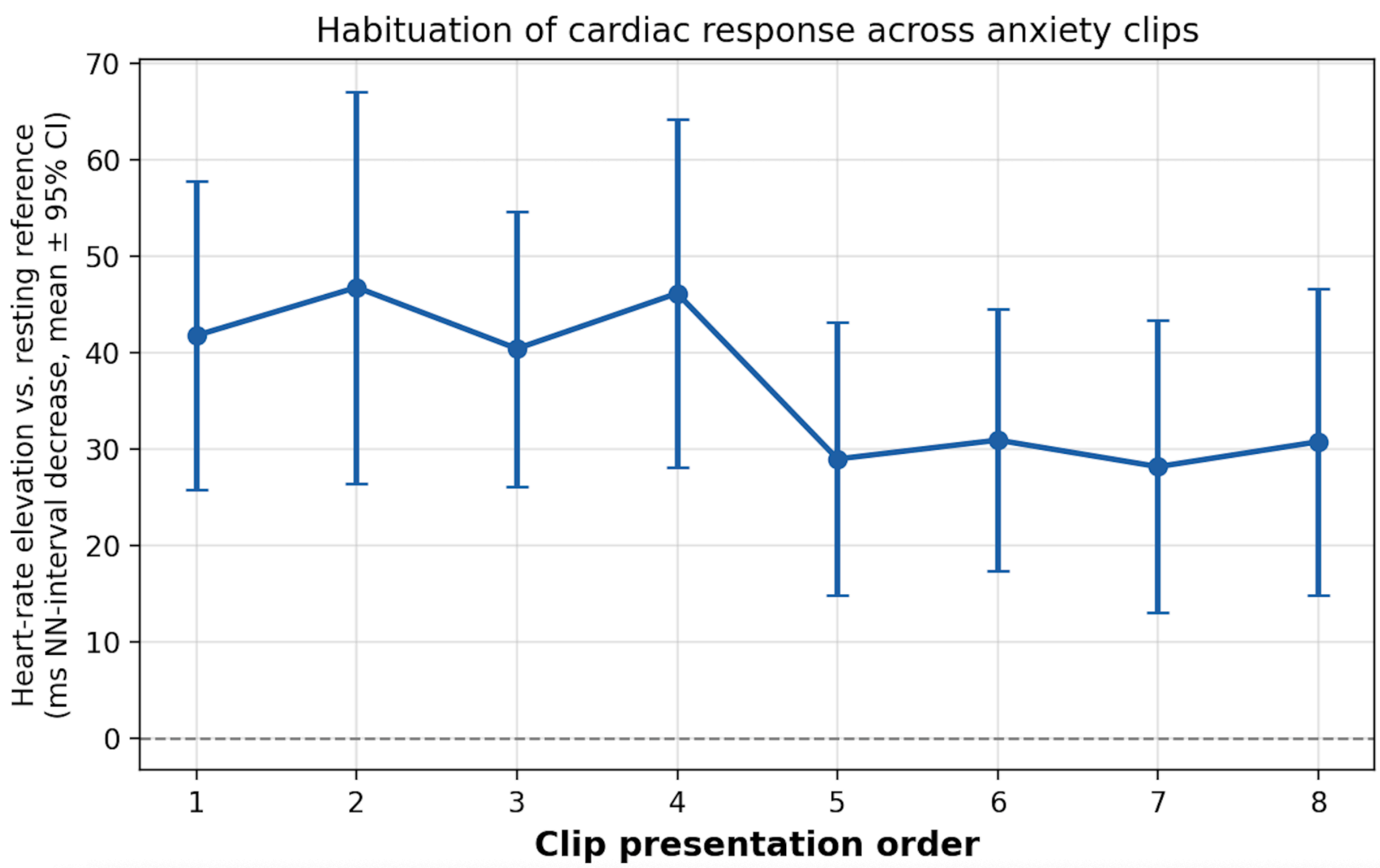

Figure S.6: Heart-rate elevation relative to the resting reference (mean ± 95% CI) as a function of clip presentation order, showing a significant decreasing trend consistent with cardiac habituation.

feature	mean_rho	p_trend	mean_early	mean_extended	mean_diff	p_early_vs_extended
**ECG_MeanNN**	-0.26889114495435307	0.0018829016779904465	42.940581166725856	32.95442972812994	9.986151438595916	0.006293680966017476
**RSP_MeanNN**	-0.032245844436772336	0.49908739466975016	158.910723917509	156.14924985659428	2.76147406091472	0.4945949924966033
**EDA_Mean**	-0.0338827838827839	0.7602629611814539	2.4984797561387855	2.0572646959872034	0.44121506015158174	0.5785377216012377

Table S.6: Physiological response magnitude across anxiety clips (n=52)
